# Hand-Arm Bimanual Intensive Therapy Including Lower Extremities in Infants With Unilateral Cerebral Palsy: A Randomized Clinical Trial

**DOI:** 10.1001/jamanetworkopen.2024.45133

**Published:** 2024-11-18

**Authors:** Astrid Carton de Tournai, Enimie Herman, Daniela Ebner-Karestinos, Estelle Gathy, Rodrigo Araneda, Anne Renders, Célia De Clerck, Seyma Kilcioğlu, Laurence Dricot, Benoît Macq, Yves Vandermeeren, Yannick Bleyenheuft

**Affiliations:** 1 Institute of Neuroscience, Université Catholique de Louvain (UCLouvain), Brussels, Belgium; 2 Exercise and Rehabilitation Science Institute, School of Physical Therapy, Faculty of Rehabilitation Science, Universidad Andres Bello, Santiago, Chile; 3 Neurology Department, Stroke Unit/Motor Learning Lab, Centre Hospitalier Universitaire UCLouvain Namur, Site Godinne, Yvoir, Belgium; 4 Louvain Bionics, UCLouvain, Louvain-la-Neuve, Belgium; 5 Physical Medicine and Rehabilitation, Clinique Universitaire Saint-Luc, Brussels, Belgium; 6 Institute of Information and Communication Technologies, Electronics and Applied Mathematics, UCLouvain, Louvain-la-Neuve, Belgium

## Abstract

**Importance:**

Earlier detection of cerebral palsy (CP) and the high neuroplastic potential during the first years of life have motivated a search for early interventions to improve children’s long-term motor abilities.

**Objective:**

To determine the effectiveness of baby Hand-Arm Bimanual Intensive Therapy Including Lower Extremities (HABIT-ILE) to improve motor function in infants with unilateral CP (UCP).

**Design, Setting, and Participants:**

This parallel group, 1:1, randomized clinical trial was conducted between December 1, 2020, and September 9, 2022, in infants recruited through centers specializing in CP treatment and parents’ spontaneous applications. Therapy took place in Brussels, Belgium, from March 8, 2021, through June 17, 2022. Infants were matched in pairs by age and lesion type and randomized to either the treatment or control group. Infants were assessed at baseline (T0) and 1 (T1) and 3 months (T2) follow-up. Inclusion criteria were aged 6 to 18 months at T0 (corrected age if preterm birth), a diagnosis or being at risk of UCP, and the ability to comply with the testing and training procedures. Exclusion criteria were uncontrolled seizures, botulinum toxin injections, orthopedic surgery, or specific intensive therapy within 6 months before and until the end of the study.

**Intervention:**

Infants in the treatment group received 50 hours of baby HABIT-ILE over 2 weeks, while those in the control group continued their usual motor activities.

**Main Outcomes and Measures:**

The primary outcome was use of the more affected hand as measured using the Mini-Assisting Hand Assessment (Mini-AHA). Secondary outcomes included Canadian Occupational Performance Measure (COPM) performance and satisfaction scores, Gross Motor Function Measure-66 (GMFM-66) scores, and other motor and functional outcomes. Between-group comparisons were calculated using repeated-measures analysis of variance (2 groups × 3 assessment times). Effect sizes were reported as partial η squared (η_p_^2^) (small, 0.01; medium, 0.06; large, 0.14).

**Results:**

Of the 48 infants entering the study, 46 (mean [SD] age, 13.3 [4.1] months; 27 boys [58%]) were included in the final analyses, with 24 in the treatment group and 22 in the control group. Group × assessment time interactions showed significant improvements that favored the treatment group for the Mini-AHA (mean [SE] difference from T0 to T2, 7.4 [1.4] Mini-AHA units in the treatment group vs 1.9 [1.5] Mini-AHA units in the control group; *P* = .008; ƞ_p_^2^ = 0.11) and for both parts of the COPM (mean [SE] difference from T0 to T2, 5.0 [0.4] in the treatment group vs 2.9 [0.4] in the control group; *P* < .001; ƞ_p_^2^ = 0.35 for the performance score and 4.4 [0.4] in the treatment group vs 2.1 [0.4] in the control group; *P* < .001; ƞ_p_^2^ = 0.33 for the satisfaction score). Although both groups improved in the GMFM-66 (mean [SE] difference from T0 to T2, 6.6% [0.7%] logits in the treatment group vs 5.5% [0.7%] logits in the control group; *P* < .001; η_p_^2^ = 0.68), there was no significant interaction (*P* = .43; η_p_^2^ = 0.02).

**Conclusions and Relevance:**

This randomized clinical trial demonstrates the feasibility of delivering 50 hours of HABIT-ILE over a 2-week period in infants with UCP. These findings show that the intervention is effective in improving motor abilities, as revealed by an increase in the use of the more affected hand in bimanual tasks and in enhanced reported functional goal outcomes.

**Trial Registration:**

ClinicalTrials.gov Identifier: NCT04698395

## Key Points

**Question:**

Does baby Hand-Arm Bimanual Intensive Therapy Including Lower Extremities (HABIT-ILE) improve bimanual performance of infants with unilateral cerebral palsy (UCP) compared with usual motor activities?

**Findings:**

In this randomized clinical trial of 48 infants with UCP assigned 1:1 to either baby HABIT-ILE or continued usual motor activities for 50 hours over 2 weeks, those who received the intervention showed significantly increased improvements in bimanual performance.

**Meaning:**

These findings demonstrate that administration of 50 hours of baby HABIT-ILE for 2 weeks in infants with UCP is effective in improving use of the more affected hand in bimanual activities.

## Teaser

This randomized clinical trial investigates the effectiveness of bimanual intensive therapy including lower extremities in improving motor function in infants with unilateral cerebral palsy.

## Introduction

With prevalence rates of more than 2 per 1000 live births,^1^ cerebral palsy (CP) is the most frequent cause of pediatric motor disability.^2^ Unilateral CP (UCP) affects 1 side of the body, often due to perinatal stroke or unilateral porencephalic cavities resulting from white matter damage or malformations.^1,3^ In addition, the primary brain injury in CP may initiate a cascade of secondary damage, such as chronic inflammation^4,5,6^ and altered organization and projections of the corticospinal tract (CST).^7,8,9^ These initial and secondary forms of damage manifest in motor and/or nonmotor impairments, notably with respect to gross and fine motor performance, potentially leading to restricted social participation.^2,10^

Over the past decade, the early detection of CP and growing appreciation of the neuroplastic potential of infants during the first 2 years of life have highlighted the need for early interventions.^11^ Motor training during an early therapeutic window may have a large positive impact on CST reorganization, while specifically alleviating the onset of secondary maladaptive neurologic damage following the primary injury.^12,13^ In addition, early attainment of motor skills may set the stage for musculoskeletal and nonmotor development.^11^ Early interventions may thus alleviate the secondary developmental damage and induce long-term motor improvements, thereby mitigating long-term disability.

The window of opportunity for an optimal impact on CST reorganization and myelination is estimated to be within the first 18 to 24 months of life.^8,14^ Early intensive interventions are currently under development to match the needs and abilities of infants with CP. Most of the proposed interventions have focused on the upper extremities, aiming to improve hand function either bimanually^15^ or unimanually.^16,17,18^ To our knowledge, the Hand-Arm Bimanual Intensive Therapy Including Lower Extremities (HABIT-ILE) is the only approach that focuses simultaneously on the trunk and upper and lower extremities.^19^ This child-friendly intervention, based on motor skill learning principles, has demonstrated efficacy in improving motor function in the upper and lower extremities of children with UCP^20,21,22,23^ aged 1.5 to 17.0 years of age, with notable motor improvements in toddlers (aged 1-4 years).^21,22^ It is therefore of major interest to explore the effects of HABIT-ILE in infants with UCP.

In this randomized clinical trial (RCT), we investigated the feasibility of administering 50 hours of baby HABIT-ILE over 2 weeks to infants aged 6 to 18 months with UCP and assessed its effectiveness in improving bimanual motor function at 1- and 3-month follow-up compared with usual (spontaneous and unstructured) motor activity. We hypothesized that this intensive intervention, representing, to our knowledge, the largest daily dosage of motor therapy ever provided at such a young age,^24^ would lead to larger improvements in motor function than would usual motor activities.

## Methods

This RCT was granted full ethics approval by the Comité d’éthique hospitalo-facultaire/UCLouvain. All parents provided written informed consent. This study followed the Consolidated Standards of Reporting Trials (CONSORT) reporting guideline for randomized studies. The trial protocol and statistical analysis plan are available in Supplement 1.

### Study Population

Between December 1, 2020, and September 9, 2022, 48 infants were recruited by A.C.d.T. and E.H. for this study from rehabilitation centers in Belgium and via spontaneous applications from parents through social networks. Inclusion criteria were (1) aged 6 to 18 months (corrected age if preterm birth) at first assessment, (2) diagnosis of (or at risk for) UCP, and (3) ability to comply with the needs of the testing. Exclusion criteria were (1) uncontrolled seizures; (2) botulinum toxin injections, orthopedic surgery, or specific intensive therapy within the 6 months preceding and extending until the end of the study; (3) any contraindications to magnetic resonance imaging; and (4) severe visual or cognitive impairments preventing the child from engaging in simple structured games.

### Study Design and Data Collection

The study design was a parallel group, 1:1 RCT. Matched infant pairs were randomized using a computer-generated randomization program^25^ by A.C.d.T., E.H., and D.E.-K. before the baseline assessment to either the treatment (n = 24) or control (n = 24) group. Pairs were formed by matching age (within 4 months) and lesion type (ie, brain malformation, periventricular white matter lesion, or gray matter lesion^3^). To arrive at our sample size, we applied a priority order to match the infants (age, then lesion side, then lesion type), which resulted in only 2 exceptions where the lesion type did not match within the pair. Whenever possible, we also matched the affected side. All infants were assessed 3 times: at baseline (T0), 1 month after baseline (T1), and 3 months after baseline (T2). Between T0 and T1, the treatment group received 50 hours of baby HABIT-ILE (structured motor activity), while the infants in the control group followed their usual motor activities (spontaneous, unstructured motor activity for an estimated 50 hours within 2 weeks)^21^ and otherwise continued their conventional treatment. Sessions were performed between March 8, 2021, and June 17, 2022 and video recorded for later assessment of the primary and secondary outcomes.

### Assessments

#### Primary Outcome

To assess the use of the more affected hand as assistant in bimanual activities, we chose as the primary outcome the Mini-Assisting Hand Assessment (Mini-AHA), which was scored based on the videotapes by certified examiners masked to group allocation and timing of assessments.^26^ The Mini-AHA items are scored using the logit-based Mini-AHA unit scale ranging from 0 to 100, with higher scores indicating better use of the more affected hand.

#### Secondary Outcomes

The Gross Motor Function Measure-66 (GMFM-66) was used to assess the infants’ gross motor function^27^ and scored based on the videotapes by experienced raters masked to group allocation. The GMFM-66 has been proven to be reliable and valid for assessing children from birth to 3 years of age.^28^ Similarly, the motor scale of the Bayley Scales of Infant and Toddler Development-Third Edition (BSID-III) was used to assess gross and fine motor skills.^29^

The semi-structured interview of the Canadian Occupational Performance Measure (COPM) was used to define the functional goals expressed by parents, who quantified their child’s performance and satisfaction for each goal.^30^ Functional goals are presented in eAppendix 1 in Supplement 2. The parents’ responses to the Pediatric Evaluation of Disability Inventory-Computer Adaptive Test (PEDI-CAT) questionnaire were used to evaluate the daily activities and mobility of the infants.^31^ Finally, the parents completed the Young Children’s Participation and Environment Measure (YC-PEM) questionnaire to evaluate their child’s participation.^32^ The infants’ actual daily amount of activity performed by each upper extremity (activity count^22^) was registered using inertial sensors on the wrists in both study groups (eAppendix 2 in Supplement 2).

### Procedures

#### Control Group: Usual Motor Activities

We chose usual motor activities as the control condition since infants’ spontaneous activity is oriented toward discovery of their environment, with motor activities typically occupying approximately 5 hours per day.^33^ Infants in the control group continued to attend daycare or to be cared for by their relatives, depending on their custom before inclusion in the study. They also continued their usual therapy during the trial, including occupational therapy, physiotherapy, and psychomotor therapy (mean [SD], 1.67 [0.52] hours per week).

#### Treatment Group: Baby HABIT-ILE

Baby HABIT-ILE, an adaptation of HABIT-ILE,^19,20^ was administered in a camp setting inside a building located in the middle of a private park for 5 hours a day (excluding nap breaks), 5 days a week, over 2 weeks for a total of 50 hours. To document real motor engagement time and treatment content, interventionists completed daily reporting sheets. Baby HABIT-ILE, as described in detail in the study protocol^34^ and in eAppendix 3 in Supplement 2, applies a hands-off concept, using games to improve the motor skills needed to attain the individual child’s functional goals set by the parents.

#### Sample Size

Our sample size calculation was based on the AHA score of a pilot study performed in 10 young children aged 1 to 4 years with UCP.^21,34^ In that study, there was a mean (SD) improvement of 8.2 (5.9) AHA units after HABIT-ILE. We hypothesized an incremental improvement of 1 SD between the treatment and the control groups at T1 and T2 (mean [SD] between-group difference, 7.2 [5.9] AHA units). Thus, we calculated a sample size of 15 infants per group with α = .05 and 1 − β = 0.9. Considering the potential for dropouts and that the AHA and Mini-AHA are different measures with noncomparable scales, we recruited 24 infants for each group. We did not collect data on race and ethnicity as these variables were not deemed relevant to the primary objectives of this study.

### Statistical Analysis

The data analyses were performed using SPSS, version 29.0 software (IBM Corporation). A modified intention-to-treat analysis was performed that included all available data. We calculated the between-group comparison using a 2-factor repeated-measures analysis of variance (RM ANOVA) (2 groups [control vs treatment] × 3 assessment times [T0, T1, T2]), with the RM as the time factor and Holm-Šídák post hoc analyses for pairwise multiple comparisons. We report effect size as partial η squared (η_p_^2^), which is interpreted as small (0.010-0.059), medium (0.060-0.139), or large (≥0.140).^35^ Prior to each analysis, data normality was checked using histograms and the Shapiro-Wilk test. If normality was verified, we next assessed sphericity using the Mauchly test and then applied the Greenhouse-Geisser correction to adjust data when the sphericity assumption was violated. In the absence of normality, we performed Friedman RM ANOVA on ranks separately in each group, with a Bonferroni correction. A 2-sided *P* < .05 was considered significant.

## Results

### Participants

**Figure 1.**
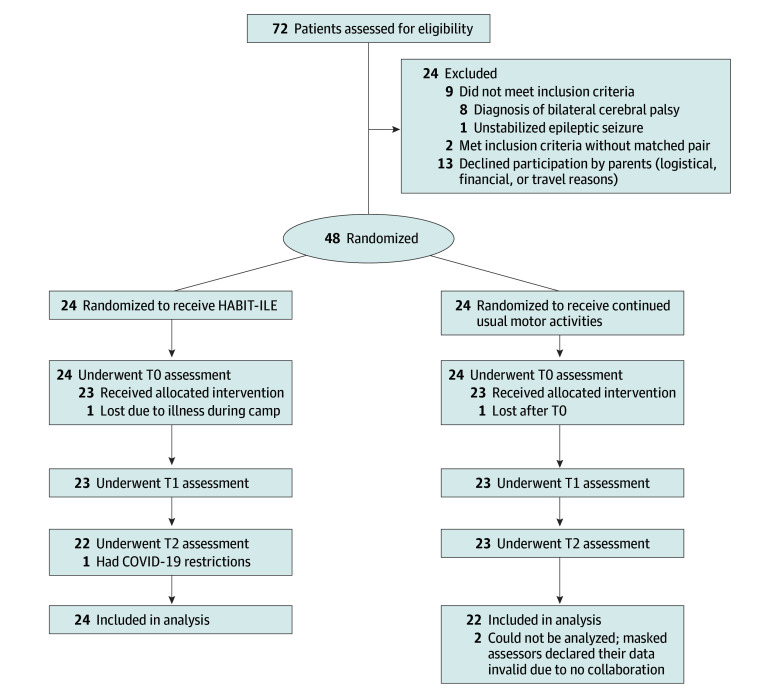
Flow Diagram of Participants HABIT-ILE indicates Hand-Arm Bimanual Intensive Therapy Including Lower Extremities; T0, first assessment time (baseline); T1, second assessment time 1 month after first assessment; and T2, third assessment time 3 months after first assessment.

Of the 72 infants screened for participation, 24 were not eligible (Figure 1). Forty-eight infants met criteria and were randomized, and 46 were included in the final analyses (mean [SD] age, 13.3 [4.1] months; 19 girls [41%] and 27 boys [58%]), with 24 in the treatment group and 22 in the control group.

Among the 48 randomized infants, 2 dropped out from the treatment group and 1 from the control group (intention to treat applied) (Figure 1; eTable 1 in Supplement 2). Two infants could not be analyzed because they did not participate in the assessments presumably because of a lack of interest associated with cognitive impairments; the masked examiners declared their data invalid. One infant from the control group did not perform for the Mini-AHA assessment at all 3 assessment times, and 1 infant in the control group did not perform for the fine motor subscale assessment of the BSID-III at T2 because he was no longer cooperative. Infants’ characteristics at baseline are presented in Table 1 and eTable 2 in Supplement 2 (characteristics at baseline and at T2 for the 46 analyzed infants).

**Table 1.** Participant Characteristics at Baseline

Characteristic	Group, No. (%)		Total (N = 48)
	Treatment (n = 24)	Control (n = 24)	
Corrected age, mean (SD) [range], mo	13.4 (4.3) [6-19]	13.0 (4.1) [7-19]	13.2 (4.2) [6-19]
Sex			
Female	11 (46)	8 (33)	19 (40)
Male	13 (54)	16 (67)	29 (60)
Lesion side			
Right	11 (46)	11 (46)	22 (46)
Left	13 (54)	13 (54)	26 (54)
Lesion type			
Brain maldevelopment	2 (8)	3 (13)	5 (10)
Periventricular	5 (21)	3 (13)	8 (17)
Gray matter	17 (71)	18 (75)	35 (73)
Prematurity			
<36 wk	7 (29)	7 (29)	14 (29)
≥36 wk	17 (71)	17 (71)	34 (71)
GMFCS-E&R level^a^			
I	5 (21)	4 (17)	9 (19)
II	7 (29)	7 (29)	14 (29)
III	6 (25)	6 (25)	12 (25)
IV	5 (21)	4 (17)	9 (19)
V	1 (4)	3 (12)	4 (8)
Mini-MACS level^b^			
I	5 (21)	7 (29)	12 (25)
II	9 (37)	4 (17)	13 (27)
III	8 (33)	8 (33)	16 (33)
IV	2 (8)	3 (12)	5 (10)
V	0	2 (8)	2 (4)

Abbreviations: GMFCS-E&R, Gross Motor Function Classification System-Expanded and Revised (aged <2 years); Mini-MACS, Manual Ability Classification System (aged 1-4 years; scored by clinicians).

^a^ Level I indicates higher gross motor function, and level V indicates more severe gross motor function limitations.

^b^ Level I indicates higher manual ability, and level V indicates more severe manual ability limitations.

### Treatment Characteristics

Among the 24 infants of the treatment group, a mean (SD) of 2785 (569) minutes of engagement time was reported, ie, 46.5 of the targeted 50 hours (93%). More information about the treatment characteristics is presented in eAppendix 4 and the eFigure in Supplement 2.

### Primary Outcome: Mini-AHA

**Figure 2.**
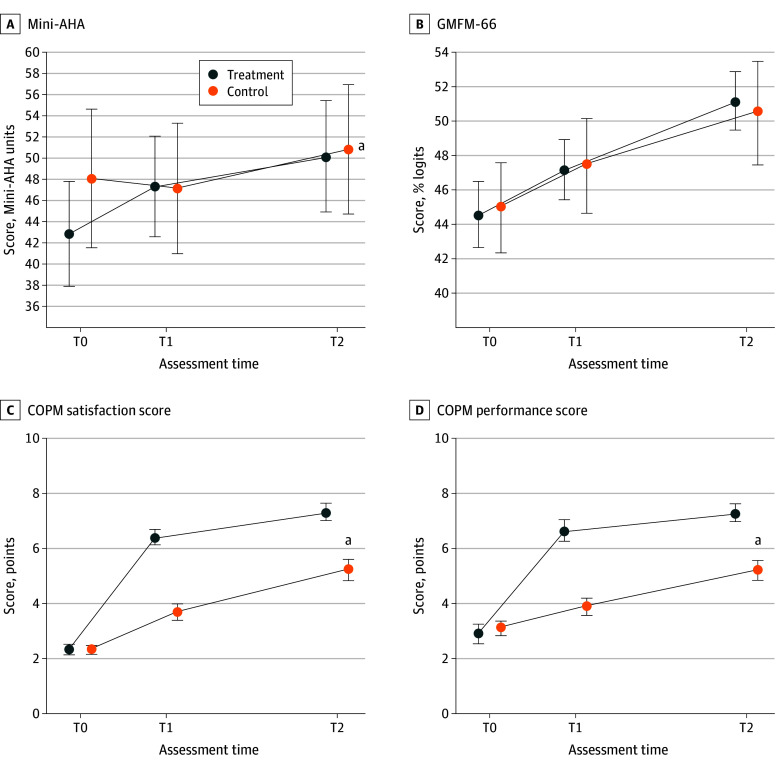
Motor Scores Over Time Dots indicate the mean values, and the error bars indicate SE. A, Mini-Assisting Hand Assessment (Mini-AHA) items are scored using the logit-based Mini-AHA unit scale ranging from 0 to 100, with higher scores indicating better use of the more affected hand. B, Gross Motor Function Measure-66 (GMFM-66) items are rated on a 0 to 100 logits scale, with higher scores indicating better gross motor abilities. C and D, Canadian Occupational Performance Measure (COPM) satisfaction and performance are scored on a scale of 1 to 10, with higher scores indicating high satisfaction and performance. T0 indicates first assessment (baseline); T1, second assessment time 1 month after first assessment; and T2, third assessment time 3 months after first assessment. ^a^Significant interaction at *P* < .05.

The Mini-AHA scores did not differ between groups at baseline (Table 2). Between T0 and T1, the Mini-AHA scores increased by a mean (SE) 4.6 (1.4) Mini-AHA units in the treatment group and decreased by a mean (SE) 0.8 (1.5) Mini-AHA units in the control group. Between T0 and T2, the Mini-AHA scores increased by a mean (SE) 7.4 (1.4) Mini-AHA units in the treatment group and increased by a mean (SE) 1.9 (1.5) Mini-AHA units in the control group. Between T1 and T2, the Mini-AHA scores increased by a mean (SE) 2.9 (1.0) Mini-AHA units in the treatment group and 2.6 (1.1) Mini-AHA units in the control group. The RM ANOVA results showed a main effect of assessment time (*P* < .001; η_p_^2^ = 0.22) and a group × assessment time interaction (*P* = .008; η_p_^2^ = 0.11) (Table 2). Post hoc tests showed improvements between T0 and T1 (mean [SE] difference, 4.6 [1.4] Mini-AHA units; *P* = .008), between T0 and T2 (mean [SE] difference, 7.4 [1.4] Mini-AHA units; *P* < .001), and between T1 and T2 (mean [SE] difference, 2.9 [1.0] Mini-AHA units; *P* = .02) for the treatment group. There were no such significant changes for the control group (Figure 2A).

**Table 2.** Primary and Secondary Outcomes (Except YC-PEM)

Assessment and group	Mean (SD)			Two-way RM ANOVA (2 groups × 3 times)			
	T0	T1	T2	Time effect		Group × assessment time interaction	
				*df*; *F* or χ^2^	*P* value (η_p_^2^)^a^	*df*; *F*	*P* value (η_p_^2^)^a^
**Mini-AHA, Mini-AHA units^b^**							
Control	48.8 (31.9)	48.0 (29.6)	50.6 (30.0)	*df* = 1.72; *F* = 12.27	<.001 (0.22)^c^	*df* = 1.72; *F* = 5.53	.008 (0.11)^c^
Treatment	42.4 (24.7)	47.0 (23.9)	49.8 (26.4)				
**GMFM-66, % logits^d^**							
Control	45.0 (12.3)	47.4 (12.9)	50.5 (14.1)	*df* = 2.00; *F* = 92.86	<.001 (0.68)^c^	*df* = 2.00; *F* = 0.86	.43 (0.02)
Treatment	44.6 (9.5)	47.2 (8.4)	51.2 (8.4)				
**COPM children’s performance score^e^**							
Control	2.3 (0.7)	3.7 (1.3)	5.2 (1.8)	*df* = 1.43; *F* = 194.50	<.001 (0.82)^c^	*df* = 1.43; *F* = 23.97	<.001 (0.35)^c^
Treatment	2.3 (0.9)	6.4 (1.4)	7.3 (1.6)				
**COPM parent’s satisfaction score^e^**							
Control	3.1 (1.2)	3.9 (1.4)	5.2 (1.7)	*df* = 1.45; *F* = 100.98	<.001 (0.70)^c^	*df* = 1.45; *F* = 21.53	<.001 (0.33)^c^
Treatment	2.9 (1.7)	6.6 (1.9)	7.3 (1.6)				
**PEDI-CAT: daily activity, scaled score^f^**							
Control	44.4 (4.3)	44.0 (3.8)	45.6 (4.4)	*df* = 2.00; *F* = 15.87	<.001 (0.26)^c^	*df* = 2.00; *F* = 2.53	.09 (0.05)
Treatment	43.2 (3.4)	44.0 (3.7)	44.8 (3.8)				
**PEDI-CAT: mobility, scaled score^f^**							
Control	49.6 (6.3)	50.7 (5.8)	52.3 (5.9)	*df* = 1.70; *F* = 24.34	<.001 (0.36)^c^	*df* = 1.70; *F* = 0.86	.41 (0.02)
Treatment	49.2 (5.2)	50.5 (4.8)	53.1 (4.3)				
**BSID-III fine motor, median (IQR), raw score^g^**							
Control^h^	29.5 (22.0-32.7)	32.0 (24.2-35.0)	34.0 (26.5-36.0)	*df* = 2.00; χ^2^ = 55.54	<.001^c^	NA	NA
Treatment^h^	30.0 (21.2-32.7)	29.5 (25.0-33.7)	32.5 (27.5-36.0)				
**BSID-III gross motor, raw score^g^**							
Control	31.8 (11.3)	34.7 (11.1)	37.4 (11.1)	*df* = 1.58; *F* = 86.55	<.001 (0.66)^c^	*df* = 1.58; *F* = 1.09	.33 (0.02)
Treatment	31.5 (9.3)	35.9 (8.1)	38.1 (8.6)				

Abbreviations: BSID-III, Bayley Scales of Infant and Toddler Development-Third Edition; COPM, Canadian Occupational Performance Measure; GMFM-66, Gross Motor Function Measure-66; Mini-AHA, Mini-Assisting Hand Assessment; NA, not applicable; PEDI-CAT, Pediatric Evaluation of Disability Inventory-Computer Adaptive Test; RM ANOVA, repeated-measures analysis of variance; T0, first assessment time (baseline); T1, second assessment time 1 month after first assessment time; T2, third assessment time 3 months after first assessment time; YC-PEM, Young Children’s Participation and Environment Measure.

^a^ Denotes the effect size (small, 0.01; medium, 0.06; large, 0.14).

^b^ Scored using the logit-based Mini-AHA unit scale ranging from 0 to 100, with higher scores indicating better use of the more affected hand.

^c^ Significant value.

^d^ Rated on a 0 to 100 logits scale, with higher scores indicating better gross motor abilities.

^e^ Rated on a scale of 1 to 10, with higher scores indicating higher performance or satisfaction.

^f^ Rated on a scale of 20 to 80, with higher scores indicating greater levels of functional ability.

^g^ Rated on a scale of 0 to 66 (fine motor) and from 0 to 72 (gross motor), with higher scores indicating greater fine and gross motor skills.

^h^ Nonparametric statistics (Friedman RM ANOVA on ranks).

### Secondary Outcome Measures

For the GMFM-66, there was a main effect of assessment time (mean [SE] difference from T0 to T2, 6.6% [0.7%] logits in the treatment group vs 5.5% [0.7%] logits in the control group; *P* < .001; η_p_^2^ = 0.68), but no significant group × assessment time interaction (*P* = .43; η_p_^2^ = 0.02) (Figure 2B; Table 2). Regarding functional goals between T0 and T2, for the COPM, both groups showed improved child performance (mean [SE] difference, 5.0 [0.4] points in the treatment group vs 2.9 [0.4] points in the control group; assessment time effect, *P* < .001; η_p_^2^ = 0.82); there was also increased parent satisfaction in the COPM ratings (mean [SE] difference from T0 to T2, 4.4 [0.4] points in the treatment group vs 2.1 [0.4] points in the control group; assessment time effect: *P* < .001 [η_p_^2^ = 0.70], respectively). However, the treatment group improved significantly more than the control group, as shown by significant group × assessment time interactions for both children’s performance score and parental satisfaction (*P* < .001 for both; η_p_^2^ = 0.35 for the control group and η_p_^2^ = 0.33 for the treatment group) (Figure 2C and D; Table 2).

The daily activity and mobility of the infants, assessed using the PEDI-CAT, showed main effects for test assessment time (daily activity: mean [SE] difference from T0 to T2, 1.6 [0.4] points in the treatment group vs 1.1 [0.4] points in the control group; *P* < .001; η_p_^2^ = 0.26; mobility: mean [SE] difference from T0 to T2, 3.9 [0.7] points in the treatment group vs 2.7 [0.7] points in the control group; *P* < .001; η_p_^2^ = 0.36). However, the daily activities domain showed no significant group × assessment time interaction (*P* = .09; η_p_^2^ = 0.05). The mobility domain also did not show significant interaction with assessment time (*P* = .41; η_p_^2^ = 0.02) (Table 2).

For the raw scores of the fine motor subscale of the BSID-III, both groups improved over time (median [IQR] difference from T0 to T2, 3.5 [2.0-6.0] in the treatment group vs 4.0 [2.0-6.0] points in the control group; Friedman RM ANOVA on ranks, *P* < .001). For the raw scores of the gross motor subscales, both groups also improved over time (mean [SE] difference from T0 to T2, 6.5 [0.8] in the treatment group vs 5.6 [0.8] points in the control group; *P* < .001; η_p_^2^ = 0.66), but there was no group × assessment time interaction (*P* = .33; η_p_^2^ = 0.02) (Table 2).

Regarding participation as assessed with the YC-PEM, there was a main effect of test assessment time for the percentage of activities and level of involvement of the home domain; the average frequency and environmental support of the daycare and preschool domain; and the average frequency, percentage of activities, and environmental support of the community domain. However, only the environmental support of the community domain presented a significant group × assessment time interaction (*P* = .005; η_p_^2^ = 0.12), favoring the treatment group (eTable 3 in Supplement 2).

The activity count did not differ between the treatment and the control groups (n = 8) for the less affected upper extremity (treatment: median, 144.0 [IQR, 125.4-155.4] activity counts per second; control: median, 139.1 [IQR, 131.7-164.1] activity counts per second; *P* = .09). Activity counts also did not differ for the more affected upper extremity (treatment: median, 101.8 [IQR, 96.4-113.8] activity counts per second; control: median, 116.2 [IQR, 107.4-122.4] activity counts per second; *P* = .09) (eAppendix 2 in Supplement 2).

## Discussion

Our aim for this RCT was to investigate the feasibility and effectiveness of baby HABIT-ILE in infants with UCP aged 6 to 18 months. Most infants successfully completed their scheduled intervention. Infants in the treatment group experienced improved performance in the use of the more affected hand during bimanual activities (Mini-AHA, primary outcome), attained functional goals (COPM, secondary outcome), and encountered an increased environmental support in the community domain of participation (YC-PEM, secondary outcome).

The baby HABIT-ILE is the highest daily dosage intervention proposed for infants aged 6 to 18 months.^24^ Despite the challenge in retaining the infants’ involvement and in managing nap times and parental separation, results confirmed that undertaking a planned 50 hours of intensive interventions over a 2-week period is practical in infants with UCP, with a mean engagement of 93% of the targeted time.

The group of infants who received baby HABIT-ILE exhibited better capacity of their more affected hand in bimanual activities compared with the control group. For the treatment group, the magnitude of changes in Mini-AHA units after baby HABIT-ILE was 7.4 Mini-AHA units within the 3-month period. The control and treatment groups both showed an increase between T1 and T2 (2.6 and 2.9 Mini-AHA units, respectively). This continuous increase is consistent with the projections proposed for the AHA by Eliasson et al,^36^ who suggested that most of the spontaneous development of the affected hand emerges during the early preschool period. This phenomenon may partly explain the differing changes observed in distributed practice vs shorter intensive periods. In the absence of other explanatory factors, the differences between groups may be explained by brain plasticity, possibly mediated by CST reorganization and myelination induced by the early intervention. This suggestion is congruent with a rodent study in which intensive motor skill training induced repair of the injured CST within a brief postnatal developmental window of opportunity.^37^ In humans, the analogous window of opportunity is estimated to fall between birth and the first 18 to 24 months of life.^7,8,38^ To our knowledge, the only other RCT reporting on children in that age range and using the Mini-AHA as the primary outcome compared bimanual therapy and modified constraint-induced movement therapy with a dosage of 1 hour a day, 7 days a week, for 8 weeks provided at home by parents.^39,40^ That study reported even higher changes than those observed here. This difference may be partly explained by the different dosage distribution and potential direct application by the parents of therapeutic advice in everyday life, thereby increasing the true stimulation time. In such a scenario, one of the components, ie, either the parents as caregivers or the distributed practice, might be more adaptable at that age. However, the samples of this study and ours were very different at baseline; therefore, designs with direct comparisons of infants with similar baseline abilities should be proposed in the future. In examining the results of other studies involving toddlers (aged 1.5-5.0 years) using the AHA, the magnitudes of change in the AHA score following the implementation of HABIT-ILE or constraint-induced movement therapy were 5.17 and 5.47 AHA units, respectively. These values exceed the smallest detectable difference, indicating that toddlers still showed large treatment effects, although there was a difference in HABIT-ILE responses between children younger and older than 36 months, thus advocating for an intervention before 36 months.^22,41^

The motor improvements obtained in this study may have had an effect on activities of daily living since the children receiving baby HABIT-ILE showed greater improvements in their functional goals compared with the control group. The retention of the functional goals 3 months after the intervention period (T2) demonstrates a successful transfer to daily life.

While our primary outcome, as well as the functional goals, presented significantly greater improvements in the treatment group compared with the control group, there was no group × assessment time interaction in other outcomes (GMFM-66, PEDI-CAT, and gross and fine motor domains of the BSID-III). Nonetheless, both groups significantly improved over time regarding these outcomes. This discrepancy may be interpreted in 2 ways: either there were natural developmental gains for all infants irrespective of the group or the instruments lacked sensitivity to discriminate between groups. For example, the first 2 domains of the GMFM-66 used in this study include only a few items such that any change therein is highly weighted.^27^ When comparing the current results of the GMFM-66 with those obtained in older children,^22^ the magnitude of changes observed here is larger than that observed in the treatment group of earlier HABIT-ILE study. No direct comparison can be done, however, using the Goals-Activity-Motor Enrichment intervention, which is an early therapy based on goals, activities, and motor enrichment,^42^ since the GMFM-66 was only performed after the intervention, thus not allowing any documentation of the magnitude of changes. When considered alongside findings of the Small Step Program,^43^ our results suggest the possibility of a comparable, spontaneous development of gross motor function at this early age.^43^ In the Small Step Program study, no significant differences in locomotion were observed between the treatment and control groups, as assessed by the Peabody Developmental Motor Scales, second edition.^44^

A previous RCT by our group in children aged 18 to 59 months demonstrated that those aged 36 months or older experienced far smaller motor improvements compared with the younger children.^22^ Thus, we concluded that at least for the 50-hour dosage, HABIT-ILE was more effective for children younger than 36 months. This conclusion led us to investigate in the present study the responses of infants aged 6 to 18 months, thereby testing the hypothesis of a window of therapeutic opportunity in infancy. Indeed, the magnitude of changes observed in the Mini-AHA and the larger changes observed in younger children of our group’s previous RCT confirm that a window of opportunity exists between ages 6 and 36 months. Of similar interest are the potential major benefits of interventions even before age 6 months for infant motor development. However, we note that the specific protocol and setting would need to be adapted to align with the unique needs of such young infants.^15^

### Limitations

Our study has some limitations. Despite our efforts to control stringently for confounding factors, our results may have been affected by the emergence of new neurologic signs during infancy. The specific context of the social distancing measures during the COVID-19 pandemic may have induced bias, given the limited socialization of infants who were relatively deprived of novel social interactions. Moreover, because parents knew that their infant may benefit from baby HABIT-ILE at the onset of the process or later, their responses to the questionnaires might not have been immune to some confirmation bias. Finally, this work raises new questions that should be addressed in future investigations. First, since the primary outcome baseline value may have influenced the response to treatment, a randomization based on the primary outcome might be more appropriate. Second, different modalities of parental involvement, including parents as therapists, should be tested. Testing such a modality may be of interest in potentially allowing families to benefit from the model most adapted for their child or themselves. This approach may help to mitigate the occasional emotional distress infants experience when separated from their parent (management of parental separation described in eAppendix 3 in Supplement 2). In addition, the influence of baseline hand function on the magnitude of change should be tested in a larger group.

## Conclusions

This RCT demonstrated the feasibility of delivering 50 hours of baby HABIT-ILE over a 2-week period in infants with UCP aged 6 to 18 months, as well as its effectiveness to improve the use of the more affected hand in bimanual activities and the mastering of the functional goals. These results reinforce the importance of implementing early intensive intervention for infants with UCP and are generally consistent with the neuroplasticity model presuming that an optimal window of opportunity exists between age 6 and 36 months for maximal benefits in motor development.

## Notes

Supplement 1.
Trial Protocol

Supplement 2. **eTable 1.** Baseline Characteristics of Lost or Excluded Participants **eTable 2.** Characteristics of the Participants Included in the Analysis, at T0 (Baseline) (n = 46) and T2 (n = 43) **eTable 3.** YC-PEM Results **eAppendix 1.** Functional Goals Set for the Entire Sample (n = 48) Grouped by Category, With the Frequency of Each Goal **eAppendix 2.** Method of Activity Count for Both Upper Limbs **eAppendix 3.** Description of Content and Organization of Baby HABIT-ILE **eAppendix 4.** Treatment Characteristics **eFigure.** Treatment Characteristics: Distribution of Motricity Types for the Upper Extremities and the Lower Extremities, Including the Trunk

Supplement 3.
Data Sharing Statement
