# Supplementary material for: Hand-Arm Bimanual Intensive Therapy Including Lower Extremities in Infants With Unilateral Cerebral Palsy: A Randomized Clinical Trial
Source: JAMA Netw Open. 2024 Nov 18;7(11):e2445133. doi: 10.1001/jamanetworkopen.2024.45133 (PMC11574690; doi:10.1001/jamanetworkopen.2024.45133)
Supplement: Supplement 1. — Trial Protocol [file jamanetwopen-e2445133-s001.pdf]

2020 call for Action de Recherche Concertée (ARC)

Fédération Wallonie-Bruxelles, Belgium

Woluwe-Saint-Lambert, November 15, 2019

In vivo estimation of the white matter microstructure in motor pathways for the assessment of motor outcome in patients with brain damage (MICROMOTO)

Proposal from "Louvain Bionics"

BLEYENHEUFT Yannick

Motor Skill Learning and Intensive Neurorehabilitation lab, COSY, Institute of Neuroscience (IoNS), UCLouvain

Department of Behavioral Sciences, Columbia University, NY, USA

MACQ Benoît

Institute of Information and Communication Technologies, Electronics and Applied Mathematics (ICTEAM), UCLouvain

VANDERMEEREN Yves

NEUR division, Institute of NeuroScience (IoNS), UCLouvain

Stroke Unit/ NeuroModulation Unit (NeMU), Neurology Department, CHU UCL Namur (site Godinne)

DRICOT Laurence

NEUR division, Institute of NeuroScience (IoNS), UCLouvain

Contents

PART I: Goals and state-of-the-art 3

Introduction 3

Grand challenges of the research 4

Grand challenge 1: A multi-facet approach for microstructure estimation from diffusion MRI 5

State of the art 5

Research hypotheses of Grand Challenge 1 9

Grand Challenge 2 – Longitudinal assessment of the microstructure in infants with cerebral palsy undergoing intensive rehabilitation training 10

State of the art 10

Research hypotheses of Grand Challenge 2 12

Methods and study design 13

Procedures/Methodologies 13

Evaluation criteria 14

Statistical analysis 15

Patient recruitment 15

Preliminary results 15

Grand Challenge 3: Longitudinal assessment of the microstructure in chronic stroke patients undergoing intensive rehabilitation training 16

State of the art 16

Research hypotheses of Grand Challenge 3 17

Methods and Study design 18

Procedures/Methodologies 19

Evaluation criteria 19

Statistical analysis 20

Patient recruitment 21

Grand challenge 4: exploring mechanisms of training-induced functional recovery through WM microstructure in very early lesions vs adult acquired lesions 22

Research hypotheses of Grand Challenge 4 22

MicroMoto: a high risk/high gain project 23

PART 2: Project Budget and Management 25

Bibliography 30

PART I: GOALS AND STATE-OF-THE-ART

INTRODUCTION

Brain lesion is the most prevalent cause of physical disability: cerebral palsy occurs in 1 out of 500 neonates (Graham, 2016) and stroke events are projected to affect 1 in 4 people over age 25 according to the World Stroke Organization[1].The consequences of brain injury are highly variable and usually include motor disorders (walking or grasping difficulties) resulting in long-term functional deficits (daily living activities such as dressing, eating, toileting ...), thereby limiting patients' autonomy.

Traditional neurorehabilitation for patients with motor disabilities consists of physical/occupational therapy sessions of less than an hour a few times a week while patients try to keep on with their activities of daily life (ADL) according to their physical capabilities. Motor training is a crucial component of neurorehabilitation as it may induce recovery through brain plasticity and thereby may partly reverse the damage at the origin of the motor impairment. It has recently become apparent that *intensive* motor training exhibits higher efficiency that traditional non-intensive neurorehabilitation, across age groups and disability levels (Bleyenheuft, Arnould, et al., 2015; Bleyenheuft et al., 2017; Bleyenheuft & Gordon, 2014; Hatem et al., 2016; Pollock et al., 2015).

We aim to show for the very first time that HABIT-ILE, an intensive rehabilitation program developed at UCLouvain in collaboration with a team at Columbia University, improves the recovery of motor control capabilities in two distinct populations in which intensive therapies have so far been very little studied: (i) newborns with CP and (ii) adults with chronic post-stroke disabilities. HABIT-ILE is likely to induce larger microstructural changes in the brain white matter (WM), in tracts not only involved in motor function but possibly in other functions as well (such as movement planning), treating the causes of the disability rather than the symptoms and potentially improving the efficiency of neurorehabilitation.

Whether in traditional or in intensive neurorehabilitation, each brain-lesioned patient should follow a program specifically tailored to their initial disability, their expected level of motor recovery and the actual chances of achieving the desired recovery, within the practical limits of a clinical context such as time, cost and personnel. It is therefore crucial to understand the nature and extent of brain damage, to identify the (un-)damaged areas that can be adaptively healed through WM plasticity, those that remain beyond recovery and those which can predict recovery before improvements in motor function occur.

Modern neuroimaging techniques, in particular diffusion-weighted magnetic resonance imaging (DW-MRI), have the potential to improve our understanding of the link between brain structure and motor recovery, especially as neuroplastic changes occur. Recent advances in brain microstructure (µS) estimation from DW-MRI obtained at ICTEAM jointly with teams at the Ecole polytechnique fédérale de Lausanne (EPFL, Prof. J.-P. Thiran) and Harvard Medical School (HMS, Prof. S.K. Warfield) offer new ways to non-invasively assess plastic changes in the damaged human WM. The theoretical promise of these advanced mathematical models of the DW-MRI signal is the characterization of the brain white matter at the micrometer level, providing indices reflective of the morphometry of axons, myelin sheaths and glial cells generically referred to as indices of the *microstructure*. The exact biological interpretation of these indices is subject to debate and validation is an ongoing quest. Nonetheless, compared with the phenomenological indices derived from the diffusion tensor imaging (DTI) model (Basser et al., 1994) traditionally used in neurorehabilitation studies, these novel approaches have the potential to provide more specific insights into the structural alterations of the brain WM µS underpinning (a) motor impairments and (b) the response to therapy, with the promise of developing more predictive biomarkers to guide neurorehabilitation.

[1] https://www.world-stroke.org/about-wso/wso-annual-report

During the two proposed randomized controlled trials (RCTs), high-quality, quantitative data will be collected in both CP infants and stroke adults before and after intensive neurorehabilitation with HABIT-ILE. First, clinical scores will be collected following well-established and validated assessments. Second, biomechanical data will be acquired. In the CP study, it will consist of optoelectronic motion capture measurements. In the chronic stroke study, it will be obtained on the REAplan® bimanual neurorehabilitation robot installed at CHU UCL Namur (Mont-Godinne). Third, neuroimaging will be performed, for both populations, on the new 3-Tesla GE MRI scanner installed at Cliniques Universitaires UCL Saint-Luc in the fall of 2019. Conventional MRI such as T1 and T2 will be supplemented with advanced DW-MRI leveraging the new scanner's capabilities. A number of mathematical models, from traditional DTI to our latest developments, will be fitted to the DW-MRI data to produce whole-brain maps of WM microstructural indices in complement to global WM connectivity maps.

This project is a unique opportunity to investigate the difference induced by intensive neurorehabilitation in the µS of the brain and to understand the specificity of changes in mature and immature brain to better target the components needed to induce neuroplasticity and functional recovery in each. Our efforts will also lead to the creation of an outstanding data set of DW-MRI signals and the related evaluations of motor capabilities, for both adults and children with acquired brain damage leading to life-long motor impairments. It will also set the foundation of a multidisciplinary platform for the development of neuroimaging tools. The global outreach of this project will therefore extend far beyond the walls of our research facilities at UCLouvain.

GRAND CHALLENGES OF THE RESEARCH

The grand challenges addressed by this project are four-fold and intrinsically multi-disciplinary:

1. The mathematical models studied at ICTEAM are based on sound biological descriptors and are likely to exhibit increased *sensitivity* to neuroplastic changes. More fundamentally, more validation is needed to determine the *specificity* of these models and what exactly they can "see" in the WM microstructure (µS). Popular models claiming biophysical specificity like NODDI may have shown increased sensitivity and prediction power than traditional DTI indices in a few cases but their specificity is still debated on theoretical grounds as well as on empirical grounds, both in animal and in human studies. The two clinical trials proposed in this project will constitute a platform to compare DW-MRI models ranging from the traditional DTI to our latest developments such as DIAMOND and Microstructure Fingerprinting. Sensitivity will be assessed via the ability to predict the motor recovery of patients and via differences between patients following intensive neurorehabilitation and patients receiving their usual treatment. Specificity will be informed by the location and extent of WM changes (e.g. in motor tracts), their nature (e.g. loss of axons, change in axonal caliber due to demyelination) and the consistency between the two age groups (infants vs adults). The best MRI-derived microstructural indices will be combined with other baseline clinical and biomechanical scores via advanced multi-agent algorithms to provide the most predictive biomarker of motor recovery.
2. Intensive motor skill training programs designed by Prof. Y. Bleyenheuft's team at IoNS in collaboration with the team of Prof. A. Gordon at Columbia University improve the motor abilities of school-age children with uni- and bilateral cerebral palsy. Traditional DTI indices have suggested that this improved motor outcome was, among others, related to a better overall quality of white matter axons in the corticospinal tract (CST), the primary pathway for producing voluntary movements. However, no DW-MRI approach has been able to test for finer structural alterations such as the myelination or the packing density of axons in the CST. In addition, the benefits of this neurorehabilitation method have not been yet evaluated in infants under 2 years old when neural connectivity undergoes particularly active development. Intensive motor training may have a far greater impact in this group and may prevent most of the brain damage causing the disability.

3. Elucidating the exact nature and evolution of defects in neural connectivity and µS occurring after stroke in adults and correlating it with motor function recovery is a pressing issue at IoNS. Stroke involving the motor system and especially the CST leave many stroke survivors with motor deficits and chronic impairments. The impact of a *unilateral* stroke on *bimanual* motor control and activities of daily life (ADL) are overwhelming, deserving special attention in the design of rehabilitation programs. Recent research on animal models and children with CP suggests that even in a chronic phase, specific interventions such as HABIT-ILE can drive large motor improvements and structural repair in the damaged CST (McKenzie et al., 2014; Sampaio-Baptista et al., 2013). Such interventions are based on intensive motor skill learning concepts (Byblow et al., 2015; Krakauer, 2015; Winters et al., 2015). However, it is unknown whether such functional changes could be obtained in adults, whether similar changes could be observed in the CST or whether these changes would be different in nature than those observed in infants. Novel DW-MRI markers provided by ICTEAM will help clarify the link between brain µS after stroke and improvement after intensive rehabilitation, headed by Prof. Y. Vandermeeren (IoNS / Stroke Unit of the CHU UCL Namur, Mont-Godinne).
4. The identical experimental settings of WP 2 and 3 allow us to directly compare WM µS baseline damage and training-induced microstructural changes between hemiplegic CP infants and adult stroke patients, both at the macroscopic level using whole-brain tractography and at the microscopic level using voxel-wise, model-based microstructural features. The deep underpinning of the differential repair mechanisms based on the initial conditions and age of the patients is likely to bring neurophysiological responses to the conditions enabling WM repair. Investigating the best responders depending on initial conditions should help better tailor treatment to each patient.

Our workplan will rely on several research hypotheses which will be explored through a common framework (data-processing-platform) coordinated by Dr. Laurence Dricot. A final clinical toolset will be established, efficiently combining neuroimaging signals and quantitative motricity signals measured clinically during rehabilitation programs. IoNS and ICTEAM have had several collaborations in the fields of neuroimaging and in the practical use of augmented reality for rehabilitation (Caby et al., 2011; Noirhomme et al., 2004) and this project is the ideal platform to foster further partnerships. All three principal investigators of this proposal are members of the Louvain Bionics group which connects researchers from science and technology, social sciences and health sciences for the development of rehabilitation tools. This research project also presents a unique opportunity to open a novel avenue of research to the neuroimaging platform at UCLouvain.

GRAND CHALLENGE 1: A MULTI-FACET APPROACH FOR MICROSTRUCTURE ESTIMATION FROM DIFFUSION MRI

STATE OF THE ART

Diffusion-weighted magnetic resonance imaging (DW-MRI) is a non-invasive tool to probe the random diffusion of water molecules in biological tissues through the external application of magnetic-field gradients of controlled intensity, duration and modulation. It is the modality of choice to study the macroscopic and microscopic, sub-voxel scale anatomy of tissues in the white matter of the brain and spinal cord.

The white matter (WM) chiefly consists of long fibers known as *axons* covered by a *myelin* sheath, usually bundled together into *fascicles*; and *glial cells* which among other important functions produce the myelin and nurture the axons. The finer study of the morphometry of axons (e.g., diameter, density, myelin thickness) and glia (e.g., mean size, fraction of voxel occupied) is referred to as *microstructure (µS) imaging* and requires elaborate mathematical models to interpret the DW-MRI measurements. *Tractography* is another post-acquisition task which consists in extracting macroscopic WM pathways spanning multiple voxels by connecting the main orientation of axons in each voxel locally (Figure 1). At a clinical resolution of a few cubic millimeters,

the vast majority of brain voxels contain multiple fascicles of axons crossing in complex interweaving patterns (Jeurissen et al., 2013; Schilling et al., 2017). The arrangement of WM axons is simpler in the spinal cord and in the corpus callosum of the cerebrum as these voxels usually feature one single fascicle of more or less coherently aligned axons.

The promise of DW-MRI is thus to reveal µS alterations which would otherwise be invisible with conventional MRI.

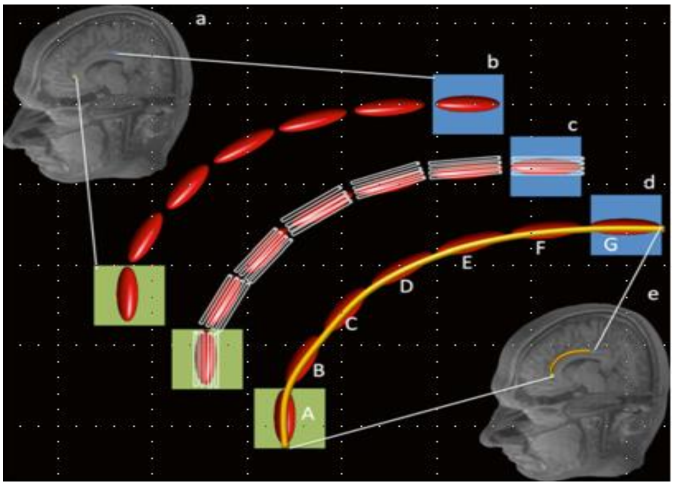

Figure 1: ***Fiber tracking or the art of drawing streamlines.*** *(a) Two voxels are selected in the brain, after whole-brain DTI estimation. (b) Visualization of the tensors estimated in relevant voxels between the two selected voxels. (c) Each tensor is a representation of the underlying white matter axons in each voxel. (d) Tractography algorithms reconstruct WM pathways by connecting neighboring fiber orientations. Figure 8.8 from* (Van Hecke et al., 2016)..

Alterations of the WM global connectivity and local µS lie at the heart of a number of motor disabilities, including in patients with stroke or cerebral palsy. Most studies of motor impairment rely on Diffusion Tensor Imaging (DTI), which has demonstrated sensitivity to µS brain damage but also suffers from a notorious lack of specificity due the global character of its mathematical formulation. Changes in DTI-derived radial diffusivity (RD) and mean diffusivity (MD) for instance may conflate gliosis and myelin clearance(Liu et al., 2013); variations in fractional anisotropy (FA) may confound axon density, myelination or axon crossings (Eaton-Rosen et al., 2015). As illustrated in Figure 2, because DTI fundamentally assumes a single population of mostly parallel axons in each voxel, it is unable to separately characterize multiple intersecting fascicles of axons, which could affect up to 90% of WM voxels at clinical resolution (Jeurissen et al., 2013; Schilling et al., 2017). In particular, DTI-based studies of the CST -a key white matter tract in motor function- are likely to be contaminated by other WM tracts such as transcallosal axons or the longitudinal fasciculus (Figure 2, bottom row).

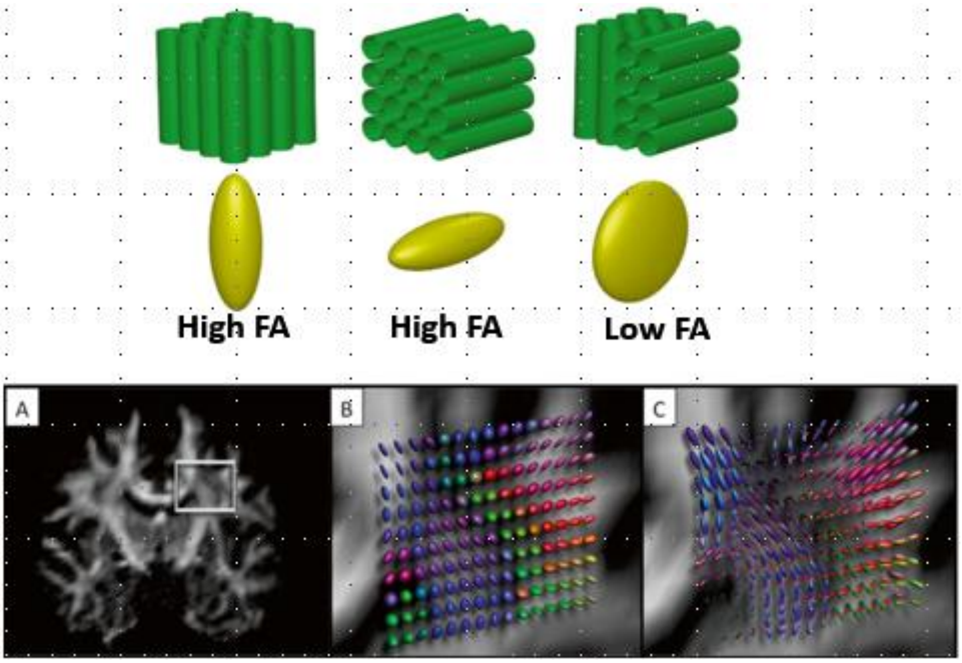

*Figure 2: **DTI cannot resolve fascicle crossings.** Top row: two fascicles of healthy axons (green cylinders) crossing each other lead to an artificially isotropic tensor and an abnormally low FA, misleadingly suggesting low "white matter integrity". Bottom row: (A) region of interest in the centrum semi-ovale where axons from the corpus callosum, cortico-spinal tract and longitudinal tract intersect. (B) DTI exhibits low-FA, isotropic tensors in those voxels. (C) A refined model (here: constrained spherical deconvolution) accounting for multiple fascicle orientations yields more biologically relevant information. Figure adapted from Figure 5.7 and 20.2 of* (Van Hecke et al., 2016).

The issue of crossing fascicles of axons was addressed at ICTEAM in connection with Harvard Medical School by extending DTI to a multi-tensor model (Scherrer et al., 2013; Taquet et al., 2012). WM connectivity and µS differences were found between patients with autism and age-matched controls (Taquet, Scherrer, Commowick, et al., 2014; Taquet, Scherrer, Peters, et al., 2014). However, as each fascicle was described by a diffusion tensor, the fascicle-specific indices still suffered from the lack of biophysical specificity inherent to DTI. More elaborate approaches known as diffusion compartment imaging (DCI) aimed at going beyond the diffusion tensor formalism with detailed biophysical modeling of the tissue. DCI models enable the separate modeling of the intra-axonal and the extra-axonal space within each fascicle of axons, which may lead to indices and biomarkers that are physically more interpretable than traditional DTI indices (e.g., axonal or glial density instead of a more abstract "diffusivity").

Sensitivity & specificity in microstructure imaging: an illustration with NODDI

The NODDI model (Zhang et al., 2012) has become popular in neurological research over the last few years (Caverzasi et al., 2016), mainly owing to the availability of a free and easy-to-use Matlab (MathWorks, Natick, MA) toolbox. NODDI is an instance of a DCI model and claims to provide indices of axon/dendrite density (ND) and axon orientation dispersion (OD). However, from a theoretical standpoint, the physical assumptions in the model have been questioned (Jelescu et al., 2016; Jelescu & Budde, 2017; Lampinen et al., 2017). In order to keep the model complexity tractable, NODDI ignores crossing fascicles and assumes one main fascicle orientation, it represents axons as "sticks" with zero radius and it fixes the diffusivity of water in the extra-axonal space in a simplistic way. When some of these hypotheses are not met, the estimated microstructural indices may change dramatically simply due to the mathematical formulation of the model [Jelescu16], which is likely to bias the interpretation of experimental results. From an experimental standpoint, the biological specificity of NODDI indices has also been challenged. In a rat spinal cord study of Wallerian degeneration conducted by ICTEAM and LDRI, NODDI predicted a significant *increase* in axon density (ND) while histological observations clearly suggested a *loss* of axons (Rensonnet et al., 2019). In a study of 2 patients a few days after

stroke, no change in traditional DTI-derived indices was observed between the lesioned and the contralateral parts of the brain (Adluru et al., 2014). NODDI on the other hand did capture changes as the ND and OD were much *higher* in the stroke area; however the biological interpretation of these two changes was not straightforward (Adluru et al., 2014). In a neuroimaging study of 10 children with unilateral cerebral palsy (Nemanich et al., 2019), while the ND was *lower* in the lesioned CST, as may be expected from the known pathophysiology (see WP 2), the lower OD was more difficult to interpret and was likely a confound of increased extra-axonal space in the WM. NODDI did exhibit better sensitivity than DTI as manual motor function was strongly correlated with the NODDI-derived ND, but not with the FA from DTI (Nemanich et al., 2019). This was likely attributable to its modeling of the diffusion in the tissue which, although imperfect, is more advanced than that of DTI. In the absence of a clear consensus on the biological interpretation of NODDI indices, care should be taken when analyzing brain maps of NODDI indices in human studies where histological verification is not possible.

<u>The quest for higher sensitivity and specificity</u>

DIAMOND, another DCI model co-developed by ICTEAM and HMS (Scherrer et al., 2016, 2017), was unlike NODDI designed for multiple intersecting fascicles. Each fascicle was assigned a set of phenomenological indices describing the diffusion of “packets” of water molecules. On the *in vivo* rat spinal cord model of unilateral Wallerian degeneration described above, DIAMOND outperformed NODDI with indices sensitive, although not unequivocally specific, to the dramatic axonal loss observed in post-mortem histology (Rensonnet et al., 2019). In addition, the DIAMOND index coined “extra-axonal restricted fraction” proved remarkably specific to microglial activation related to inflammation (Taquet et al., 2019). In a recent rat study of Wallerian degeneration induced in one optic nerve of the optic chiasm, DIAMOND indices in the degenerate nerve were markedly different from those in the intact nerve (Rojas-Vite et al., 2019). This suggested the potential of DIAMOND for characterizing the WM µS in complex areas of crossing fascicles. However, the theoretical underpinnings of DIAMOND do not provide a straightforward interpretation of its fascicle-specific indices. These indices reflect the diffusivity of “packets” of water rather than direct properties of the tissues such as axon density of axon diameter. Although far from exhaustive, validation of DIAMOND on human subjects was encouraging too. On a healthy volunteer, DIAMOND showed increased accuracy --based on the known neuroanatomy--, in the estimation of fascicles’ orientations, especially in areas of complex crossings. It extracted indices specific to the microstructure of each fascicle in a voxel which seemed consistent with the expected anatomy (Scherrer et al., 2016, 2017). In preterm infants, DIAMOND showed better sensitivity than DTI in studying microstructural maturation in the sub-cortical white matter and cortical gray matter (Eaton-Rosen et al., 2017), which supports its application to the CP population targeted in WP 2 of this project.

Our latest approach to µS estimation was dubbed Microstructure Fingerprinting (MF), co-developed with EPFL and HMS. This approach completely breaks away from the diffusion tensor formalism and from any closed-form mathematical formulations. Instead, it relies on computer simulations known as Monte Carlo to simulate the DW-MRI signal in 3D geometries representing the tissue microstructure (e.g., cylinders for axons). Depending on the 3D geometry assumed, MF provides indices directly reflecting the axon diameter distribution and the axon density in each voxel. On the in vivo rat spinal cord model of Wallerian degeneration, MF showed exquisite sensitivity to axon density (Rensonnet et al., 2019). On an ex vivo cat spinal cord, the method was able to provide accurate indices of the mean and standard deviation of axon diameters in each voxel. On a healthy volunteer of the Human Connectome Project (HCP), patterns of axon diameter and density in axons traversing the corpus callosum were found to be consistent with previous human dissection studies, suggesting the ability of the method to characterize the µS in the whole brain, including areas of crossing fascicles (Rensonnet et al., 2019).

<u>Microstructure imaging, a multi-lens microscope</u>

It is important to note that estimating a microstructural model is a post-processing analysis step and that, except for a few technical exceptions, all the aforementioned models can be estimated on the *same DW-MRI data at no additional cost* once the data has been acquired. Each model can be thought of as a lens through which some tissue features may be visible while others are hidden. This is analogous to histochemistry on two contiguous tissue slices: LFB staining of the first slice will only highlight myelin sheaths as though no other tissue component were there, while iba1 staining of the second slice will reveal microglia and seemingly ignore axons and their myelin sheaths.

While it is still unclear whether one universal model can predict all WM changes and cover all possible neuropathologies, it is likely that clinical studies will benefit from a multi-model approach both in sensitivity and specificity. In the two studies of motor impairment proposed in this project, we will acquire high-quality DW-MRI data compatible with a wide variety of DW-MRI models. Whole-brain maps of model-derived indices will be computed and will enable us to search for the best biomarkers of motor recovery, while shedding light on the microstructural processes at play in the WM, not visible on conventional MRI. Even if the exact physiological basis of those indices were not completely elucidated, establishing biomarkers that accurately predict the recovery of motor function after brain damage would be a key accomplishment for neurorehabilitation per se.

Research hypotheses of Grand Challenge 1

A multi-model approach predicts and explains motor recovery (HP1.1)

The DW-MRI data acquired in the populations studies of WP 2 and WP 3 will be of the “multi-shell HARDI” type (Alexander, 2008; Tuch et al., 2002), which is compatible with almost all models of the µS available in the literature, from the limited DTI model to our latest frameworks. A minimal list of models which will be over the whole brain will at least include the usual DTI, the popular NODDI and our DIAMOND and MF approaches, all of which can be estimated using free, open-source software. More models can be included at no additional cost. For instance, the DIPY open-source project enables users to fit up to 20 models[2]. The recent-released DMIPY toolbox[3] provides a do-it-yourself framework to build existing and new models from simple compartments used as building blocks. Some of these models should tentatively reveal fine microstructural alterations of interest in motor recovery. These include changes in axonal diameter linked to demyelination or axon density (as expected in CP), inflammation, the presence of vesicles filled with liquids or microbleeds due to leukoaraiosis (possibly associated with chronic stroke).

A multi-modal approach predicts and explains motor recovery (HP1.2)

The acquisition of data from multiple modalities lies at the core of this project. Patients undergoing neurorehabilitation will be evaluated using well-established and quantitative clinical scales. They will also be subject to biomechanical recordings either through a camera motion capture system (WP 2) or using a specifically-designed neurorehabilitation robot (WP 3). Each patient’s trajectory will therefore be thoroughly documented with a massive amount of data which our team at ICTEAM will mine using the latest advances in data analysis. Indices pertaining to different feature spaces (clinical scores, biomechanical measurements and neuroimaging) collected before treatment will complement each other to provide biomarkers able to quantify patients’ motor and function recovery.

A multi-scale approach predicts and explains motor recovery (HP1.3)

[2] https://dipy.org/tutorials/

[3] https://github.com/AthenaEPI/dmipy

The WM motor tracts or the most immediate candidate regions to look at for predicting motor recovery at baseline and for the observations of WM changes during neurorehabilitation. As such, natural analyses will include looking at local, voxel-scale μS indices in and aggregated over the CST of the damaged hemisphere, the CST of the healthy hemisphere and differences or ratios between the two.

However, other regions of white matter not directly related to the motor command but rather involved in cognitive tasks may inform motor recovery too. The pre-frontal cortex for instance is involved in the planning of movements and the posterior parietal cortex receives somatosensory, proprioreceptive and visual inputs to determine the position of one's own body and the target of the movement to be made. The salience network and its attention-control system have been shown to be critical for motor control and dexterity (Rinne et al., 2018a). Seeding points will therefore be placed at these various cortical locations to reconstruct the appropriate WM tracts using tractography. Microstructural indices will be collected from all the voxels traversed by the tractography streamlines originating from these seeds.

Finally, more diffuse damage across the whole brain WM may also affect the perspectives of motor recovery. For instance, the small-vessel disease (SVD) can exhibit stroke symptoms with no visible lesion and can turn, in the chronic phase, into normal-appearing MRI with few symptoms and a steady cognitive decline (Wardlaw et al., 2013). SVD is characterized by leukoaraiosis, i.e., microbleedings spread across the brain, which may be captured by DW-MRI μS indices. Significant changes in μS indices in areas "far away" from those associated with motor control and planning will bring about important knowledge in our understanding of the pathological processes leading to motor recovery.

We expect the biomarkers of motor recovery combining μS information at these various spatial levels of the WM to be more powerful and predictive than biomarkers obtained from indices solely in the CST.

A multi-population approach predicts and explains motor recovery (HP1.4)

The novel intensive HABIT-ILE therapy will be tested in two very different patient populations, namely infants with CP and adults in the chronic phase of stroke. Both populations will be imaged on the same scanner at St-Luc. The parameters of the diffusion gradients (duration, separation, intensity, waveform, orientation) and the distribution of these gradients across multiple "shells" (multi-shell HARDI) will be identical for both populations. This implies that any measured difference in DW-MRI signals arises solely from the WM tissue, which forms microstructural barriers to the diffusion of water. In other words, DW-MRI should provide a common scale to examine two seemingly different populations. The first tests on the new MRI scanner assisted by the GE-appointed MR physicist will determine whether the imaging parameters such as matrix and voxel size will be kept constant as well. At a fixed resolution, the motor tracts of infants will contain fewer voxels than those of adults given the overall size difference. Those tests will need to strike the right balance between sufficient resolution, signal-to-noise ratio and similarity between the two studies. In theory, microstructural indices derived from the advanced models described above are *not affected* by the voxel size because they provide indices that are averages over all the axons and cells of the voxel. At large voxel sizes however, several macro-environment may coexist and ultimately affect μS indices. Overall, we expect variables that are predictive for infants to be predictive for adults too, and conversely.

Grand Challenge 2 – Longitudinal assessment of the microstructure in infants with cerebral palsy undergoing intensive rehabilitation training

State of the art

Over the past century, significant progress has been made to eradicate many childhood diseases such as poliomyelitis, measles or diphtheria (Van Panhuis et al., 2013). However, clinical progress on how to treat cerebral palsy, the most prevalent pediatric motor disability, is lagging far behind. Cerebral palsy (CP) is

described as a group of disorders of movement and posture due to non-progressive brain damage in the developing brain (Graham, 2016). Depending on the location of the initial brain damage, children can present either a unilateral (40%) or a bilateral (60%) motor impairment (Krägeloh-Mann & Cans, 2009). These motor impairments generally lead to limitations both in gross motor function and in manual abilities, which may greatly impair the execution of many everyday life activities (e.g., getting dressed, walking, eating autonomously, etc.). These limitations impact both the lifelong autonomy and the quality of life of these children.

As the initial brain damage is considered irreversible and non-evolutive, the management of CP is currently solely focused on the treatment of the symptoms. Neurorehabilitation programs have traditionally followed neurodevelopmental approaches (e.g. Bobath, usually a few hours a week, combining stretching and movements manually guided towards "normality"), the efficiency of which is strongly debated (novak et al., 2013). More recently, there has been strong evidence that intensive interventions based on motor skill learning are more efficient in inducing functional and neuroplastic changes, especially in children over 6 years old. These "hands-off" therapies are organized as blocks of training using motor learning concepts (i.e. practice specificity, context of learning, feedback, speed–accuracy trade-off, transfer of learning, etc.), aiming to elicit practice-induced brain changes arising from repetition, increasing movement complexity, motivation and reward. Though efficient, these interventions remain limited to the mere treatment of symptoms of CP.

Recent research using animal models suggests that intensive motor skill training in infants with CP may significantly reduce or even prevent maladaptive neuroplastic changes consecutive to the brain lesion, thereby addressing some of the root causes of CP. More specifically, the initial brain damage induces a cascade of secondary neuroplastic damage which is responsible for the motor disability, including:

1. chronic inflammation (Baud et al., 2004; Favrais et al., 2011; Van Steenwinckel et al., 2014) which induces a deficit in white matter myelination. Myelin regulates axonal conduction velocity and thus the timing of information flow between different brain areas, including in the corticospinal tract (CST) which connects the cerebral cortex to the spinal cord, and which is the primary pathway for producing voluntary movement;
2. alterations in the organization of the corticospinal pathway. In normal brain development, CST axons connecting a cerebral hemicortex to the same side of the spinal cord are pruned, resulting in a crossed organization where each hemicortex solely projects to the opposite side of the spinal cord, thereby controlling the opposite hemibody (Eyre et al., 2001; Staudt, 2010). Following early brain damage in CP, direct uncrossed axons are preserved between the injured hemicortex and the more affected hemibody, which can thus be controlled by both sides of the brain (Guzzetta et al., 2007a). Studies in children over 6 years old have highlighted larger motor deficits in children with such reorganization;
3. underdeveloped corticospinal projections at the spinal level. As the CST projections to the spinal cord depend on motor stimulation during a specific developmental window, the lack of movement in paretic limbs induces an unbalance in the competition between CST terminals and other spinal neural systems (Martin, 2005; Martin & Lee, 1999).

Recent studies using animal models of CP demonstrate that this secondary damage is activity-dependent. Rodent studies have shown that motor-skill learning increases white matter DTI-derived fractional anisotropy (Brandão et al., 2018; Taub et al., 2004), suggesting that functional improvement of the CST is training-dependent (McKenzie et al., 2014; Xiao et al., 2016). In kittens, intensive motor skill training of the impaired forelimb in a specific developmental window of opportunity causes a rebalance in the CST synaptic projections to the spinal cord (Friel et al., 2012). In humans, the therapeutic window during which these projections might be rebalanced through motor stimulation has not been exactly identified. It is generally assumed to occur during early infancy, probably until the age of two (Martin & Lee, 1999), during the main period of axonal myelination in the CST (Li & Martin, 2000). In addition, transcranial magnetic stimulation (TMS) studies of motor-evoked potential in babies suggest that the CST system is organized during the first eighteen months of

life (Eyre et al., 2001), thus offering a therapeutic time window during which early stimulation may positively impact CST reorganization. Taken together these observations from animal and human studies suggest that early motor skill learning based interventions improve CST projections, thereby preventing secondary motor damage.

Compared to the animal literature, only a few motor intensive intervention trials have been carried out in very young children. To the best of our knowledge, these studies have all focused on children with unilateral CP and evaluated modified forms of constraint-induced movement therapy (CIMT), an intensive intervention performed sitting, requiring the physical immobilization (cast, glove,..) of the non-affected arm to promote the use of the affected arm (Deluca et al., 2012; Eliasson et al., 2011, 2018; Nordstrand et al., 2015; Taub et al., 2004). These studies have provided moderate support in favor of CIMT and suggested that higher daily intensity of training (Deluca et al., 2012; Taub et al., 2004) induced larger effects than lower daily intensity training (Eliasson et al., 2011, 2018; Nordstrand et al., 2015). CIMT focuses solely on the upper extremities of children with unilateral CP (Deluca et al., 2012; Eliasson et al., 2011, 2018; Nordstrand et al., 2015; Taub et al., 2004), despite the fact that the lower extremities are also commonly affected. Moreover, none of these studies has investigated the possible impact of these interventions on myelination, organization and projections of the CST, which may be accessed using advanced analyses of DW-MRI acquisitions.

RESEARCH HYPOTHESES OF GRAND CHALLENGE 2

In Grand Challenge 2, we will specifically target this secondary damage in infants with CP, aged 6 to 18 months, by demonstrating **for the first time** that it might be **prevented** by an intensive treatment program, the 'Hand-arm-bimanual-intensive-therapy including lower extremities' (HABIT-ILE). We will investigate therapeutic outcome through a multimodal analysis of neuroplastic changes (see Grand Challenge 1), a thorough assessment of motor function and activities outcomes. This will also advance our understanding of motor control changes through muscles activation and kinematic assessment. Targeting the secondary damage will in turn prevent part of the motor disability and thus improve functional abilities and motor control. For the first time, a large-scale study will be conducted to test this hypothesis in young children with unilateral CP.

We propose an evaluation of the effects of HABIT-ILE in 6-18 month old infants based on a comprehensive functional, neurological and biomechanical assessment. HABIT-ILE is an intervention based on motor skill learning principles which focuses on the bimanual use associated to postural and/or gross motor stimulation (Bleyenheuft & Gordon, 2014). Its efficiency has already been demonstrated for both upper and lower extremities in children with unilateral (Bleyenheuft, Arnould, et al., 2015) and bilateral CP (Bleyenheuft et al., 2017) between 6 and 18 years old. **We aim to show for the first time that HABIT-ILE prevents secondary motor damage in 6-18 month old infants by treating one of its causes.**

We aim to demonstrate the effectiveness of HABIT-ILE in preventing secondary damage in CP children. More specifically, we will test the following research hypotheses. In infants with unilateral CP:

1. the HABIT-ILE intervention induces superior, clinically meaningful gains on both activity limitations (primary functional outcome) and impairments (**HP2.0**)

2. The HABIT-ILE intervention induces an increase in myelination evidenced by a change in white matter microstructure integrity of the CST and cortico-cortical connections as measured from DW-MRI (primary imaging outcome), a decrease in brain inflammation, an increase in CST projections to the spinal cord, and prevents CST reorganization in the affected hemi-cortex of infants with unilateral CP (**HP2.1**)

3. the changes in WM-µS integrity correlate with mid-term changes in clinical outcomes (follow-up) **(HP2.2)**

4. the functional gains will be predicted by baseline WM-µS integrity **(HP2.3)**

5. therapy onset in the course of infants development might influence long-term neuroplastic and functional changes, earlier onset might induce larger changes **(HP2.4)**

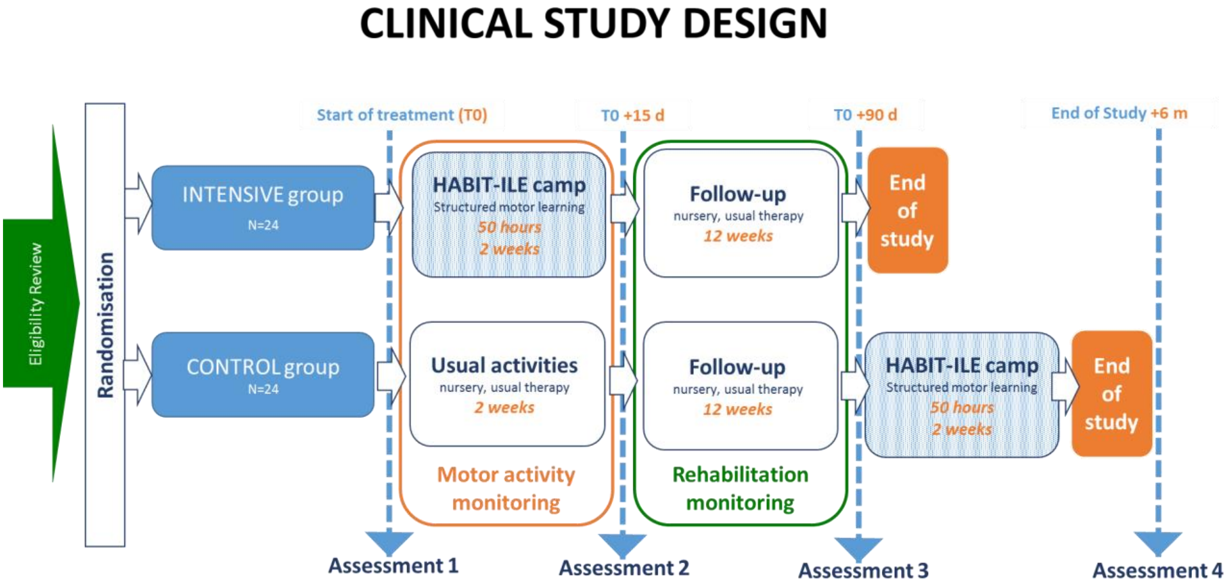

Figure 3: Design of the randomized controled trial for a population of infants with cerebral palsy.

METHODS AND STUDY DESIGN

A randomized trial (RCT) is at the core of Grand Challenge 2, involving **48 infants** with CP between 6 and 18 months. Inclusion criteria will include: being diagnosed or considered at high risk of unilateral (RCT1) cerebral palsy, 6 to 18 months inclusive and ability to complete testing according to the age. Exclusion criteria will include epilepsy, botulinum toxin injections or orthopedic surgery within the past six months or during the study period, as well as severe visual and cognitive impairments preventing the child from being interested in simple games.

A pair-matched randomization will be used. Group allocation to either HABIT-ILE or a control condition will be randomly determined within each pair. As depicted in Figure 3, the HABIT-ILE group (n=24) will receive 50 hours of treatment over 2 weeks (5 h/day, 5 days/week) whereas the control group (unilateral n=24) will follow their usual activities for 2 weeks (nursery, ongoing therapy, generally a few hours of neurodevelopmental therapy per week, etc.). Usual activities have been chosen as control intervention since spontaneous activity of infants is oriented towards the discovery of their environment with motor activities in a nursery reaching around 5 hours per day. The between-group comparisons will be carried out between T0 and T+15 and between T0 and T+90. Participants in the control group will be offered HABIT-ILE training after assessment period 3. A supplementary assessment of this group at T0+180d will allow us to investigate the influence of the onset of therapy time and persistence of the therapeutic effects on the long term.

Each assessment will comprise clinical outcomes, biomechanical assessment, and neuroimaging outcomes (traditional and multi-shell HARDI DW-MRI).

PROCEDURES/METHODOLOGIES

The intensive motor skill training consists of an adaptation of HABIT-ILE. This intervention, which we developed initially for children with CP over 6 years old, is carried out in a camp setting, with structured tasks of increasing motor difficulty and functional activities that require the use of both hands whilst sustaining postural activity or activities of the lower extremities. The therapy, using games and functional activities, is delivered in a child-

friendly environment. The tasks are chosen according to individualized functional goals previously defined by the parents (e.g. drinking or sitting autonomously, playing without help, etc.).

Although the basic principles of HABIT-ILE will be followed (Bleyenheuft & Gordon, 2014), the toys, tasks and activities will be adapted to induce spontaneously bimanual use. The duration of activities as well as lower extremities/postural associations will also be adapted to each infant depending on his/her functional objectives and personal characteristics (motor and attentional level, for instance). Eight to ten infants will undergo rehabilitation during one intensive session of 2 weeks; each infant will have a full-time therapist dedicated to his/her treatment. The follow-up of the guidelines and the fidelity to the treatment principles will be ensured through the supervision of all interventions by the post-doctoral coordinator dedicated to the project. As 50 to 60 hours of intensive therapy are needed to induce long-term motor changes (Brandão et al., 2018; Sakzewski et al., 2015), this project, in agreement with the usual motor activity time (Van Cauwenberghe et al., 2011) and the need for infants to rest, includes 5 hours per day of therapy with the same therapist for a total of 50 hours. HABIT-ILE for infants has been tested in a pilot study (illustrations in section "pilot results") which has confirmed previous evidence that this therapy intensity is both practically feasible and efficient for children that age (Taub et al., 2004).

The time-equivalent control period consists of the child's usual activity, including nursery, usual rehabilitation, etc. Both the content of this activity (notebook) and the time of activity (wearable sensors used in both groups to monitor the amount of movements) will be documented. This approach enables the comparison of the structured aspect of motor activities in therapy compared and "spontaneous activities" to be carried out.

The MRI procedure will consist in nap or night MRIs. Infants will undergo a standardized gradual adaptation program to make them accustomed to the very specific conditions of the MRI environment, including the wearing of ear-plugs previous to the scanning nights, bottle feeding just prior to the MRI scan, a careful examination of each infant's sleep routines, etc (Dean et al., 2014).

EVALUATION CRITERIA

Primary imaging outcomes (CST integrity measured with DTI, microstructures and myelination maps) will be measured at T0, T0+15 days and T0+90 days. We will use the classical DTI indicators of fractional anisotropy (index of fiber coherence and integrity), radial diffusivity (index of axon diameter and myelination), axial diffusivity, and mean diffusivity. These measures have been successfully used to demonstrate structural modifications of the CST, and provide an estimation of the fiber quality (Krägeloh-Mann & Cans, 2009). In addition we will use new advanced mathematical models which provide the opportunity to separately characterize fascicles of axons in areas of the brain where multiple white matter tracts intersect, allowing thus to better identify fibers of the CST from other crossing tracts, quantify CST projections, localize brain inflammation and detect axonal loss (Scheck et al., 2015). Tractography will be used to highlight potential macroscopic changes in CST organization (Bleyenheuft, Dricot, et al., 2015). Myelin maps will be used to assess global changes in myelination (Ganzetti et al., 2014). In addition, morphometric analysis (cortical thickness, cortical folding and sulcal depth) based on conventional T1 imaging will be used to investigate potential changes in grey matter (Remer et al., 2017). Resting state fMRI will also be used to explore the potential changes in brain's functional organization and connectivity (Wen et al., 2019). All MRI data will be analyzed by an MRI specialist unaware of the treatment allocation. Although some of these measures have already been performed in infants, notably in premature babies (Deoni et al., 2015; Eaton-Rosen et al., 2017; Remer et al., 2017; Wen et al., 2019), they have never documented potential neuroplastic changes before and after an intensive intervention.

Clinical outcomes include functional changes at the 3 levels of the International Classification of Functioning, Disability and Health (ICF): i. the **body level**, assessment of movement control, both movement pattern and quality, through **biomechanics** (i.e. electromyographic and kinematic short assessments using an optoelectric motion capture system in a task designed for infants (Ransburg et al., 2017)), and assessment of sensory processing through the Sensory Profile 2 questionnaire (Dunn & Westman, 1997; Provost & Oetter, 1994); ii. the **activity level** (gross motor function using the Gross Motor Function Measure (GMFM-66) (Russell et al., 2010), and manual ability using the Mini-Assisting Hand Assessment (mini-AHA) (Greaves et al., 2013); and, iii.

functional skills and **participation level** (PEDI-CAT (Kramer et al., 2016) , YC-PEM (Khetani et al., 2015) and the Canadian Occupational Performance Measure (Dedding et al., 2004)). All physical assessments will be videotaped and blindly scored. These clinical assessments will allow us to document whether functional abilities of the infants are improved after intervention, their nature and extent, and their correlation with neuroplastic changes. Although the number of assessments might seem quite high for children of this age, the estimated time to carry out the full assessment is less than 2 hours for the functional assessment, 30 minutes for MRI and 30 minutes for 3D movement analysis. The primary clinical outcome will be the mini-AHA. The other tests spanning the 3 ICF domains will be used as secondary clinical outcomes.

STATISTICAL ANALYSIS

No DTI pre-post intervention analyses in infants have been published so far. However, since CST integrity measured with DTI is highly correlated with manual ability (Bleyenheuft et al., 2007), we performed a sample size calculation based on the AHA score of the pilot study performed in 10 infants with unilateral CP (see "pilot data"). A mean improvement of 8,2 AHA units (standard deviation=5.9 AHA units) after the intensive therapy was reported. Thus, our hypothesis is an incremental improvement of 1 SD between the HABIT-ILE group and the control group (between group difference =7,2, SD=5,9). With $\alpha=0.05$ and a $1-\beta=0.9$, a sample size of **15 participants per group is required**. Considering potential drop-outs and data loss in MRI, **24 participants will be included in each group**. Neuroplastic changes and secondary outcomes will be compared between groups using analysis of variance (ANOVA). Non-parametric methods will be performed whenever ANOVA assumptions are not met. Time-onset influence will be tested at the 4th assessment using an ANOVA.

Separate generalized linear models (GLM) will be estimated for the CST and whole-brain WM (ICTEAM models) to infer the correlations between µS integrity at baseline/after intervention and the MSkL outcomes (bimanual SAT). Finally, an overall GLM will be estimated to combine CST and whole-brain µS integrity. The *R* software will also be used for bimanual MskL curves modelling.

PATIENT RECRUITMENT

We will recruit patients through the network of national centers for cerebral palsy, which has been active for many years. Yannick Bleyenheuft actively collaborates with the reference center of UCLouvain (Cliniques Universitaires UCL St-Luc/CHU UCL Namur (Mont-Godinne)) and with the CIRICU (HUDERF/ERASME/UZ Brussel/CHL La Citadelle/Ziekenhuis Inkendaal).

PRELIMINARY RESULTS

Grand Challenge 2 is based on strong preliminary evidence derived from pilot data collected in 10 pre-school children (12 to 40 months). All children of this pilot study successfully participated in a 2-week HABIT-ILE training camp (5 h/day over 10 days). The manual ability and functional objectives improved largely during the intervention while they did not during the 2-week control phase (see Figure 4). The assistance provided by the affected hand to the non-affected hand during bimanual activities (mini-AHA) improved by 8 %, exceeding all previous interventions targeting infants (generally in 5% over a larger time window) (Eliasson et al., 2018). Finally, the functional objectives defined with the parents showed a large progression during HABIT-ILE with results maintained at 3 months follow-up. These results demonstrated the feasibility of applying HABIT-ILE in infants and suggest that the expected functional changes might be larger than those of previous therapies in this age group. Not only should this project solidify those preliminary results but it should also shed light on the physiological inner workings of the therapy by means of novel MRI indices of WM µS and plasticity.

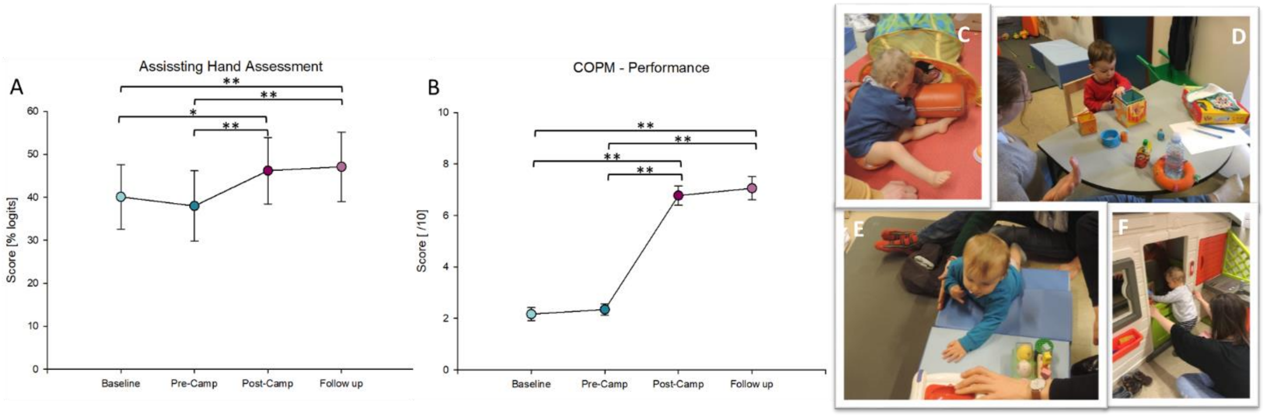

Figure 4: A, B improvements during the HABIT-ILE training. Circles=mean (+SD) of the pilot group. Panel A: assisting Hand Assessment (AHA-Units, %); Panel B: Canadian occupational performance measure (raw units); Panel C to F illustration of children aged 12 months learning during HABIT-ILE how to sit by themselves (C), use the more affected hand to manipulate (D), crawl (E) and stand independently (F) with motivating games/activities.

GRAND CHALLENGE 3: LONGITUDINAL ASSESSMENT OF THE MICROSTRUCTURE IN CHRONIC STROKE PATIENTS UNDERGOING INTENSIVE REHABILITATION TRAINING

STATE OF THE ART

Continuous medical progresses such as thrombectomy and thrombolysis gradually improve the management of acute stroke, leading to higher rates of stroke survivors. Despite advances in the care of acute stroke, many patients suffer from long-term disabilities. Among these stroke survivors, one of the most common impairments is hemiparesis which is characterized by a weakness and a loss of control on voluntary movements in the contralesional side of the body. Hemiparesis can lead to loss of independence for activities of daily life (ADLs). Stroke has thus become the primary cause of chronic disability in the Western World (Dewilde et al., 2017; Mozaffarian et al., 2016). Beyond the acute phase, a large proportion of stroke survivors (30-66%) suffer from chronic impairments (Bonita & Beaglehole, 1988; Hackett et al., 2000; Hardwick et al., 2016), with an approximate 40% dependence for daily life activities (ADL) (Hankey et al., 2002). Motor impairments, mostly hemiplegia and hemiparesis, are especially frequent and have a major impact on independence and the ability to resume work (Bonita & Beaglehole, 1988; Hankey et al., 2002; Stinear, 2010).

Post-stroke, neurorehabilitation aims to restore independence of the patient, ideally through full recovery of impairments, activity limitations and ultimately participation restrictions. Stroke-induced impairments of the upper limb (UL) (paresis, hypoesthesia, loss of motor control, spasticity …) may impede unimanual ADL, but this can be (partly) compensated by the adaptive recruitment of the non-paretic UL. In contrast, such strategic compensations cannot be used to rescue impaired bimanual ADL (Kantak et al., 2016; Maes et al., 2017). As discussed in Grand Challenge 2, recent literature on animal models and children with early brain lesions causing CP suggest that specific interventions based on intensive motor skill learning concepts might improve the quality of the damaged white matter fibers, which consequently leads to large motor changes (McKenzie et al., 2014; Sampaio-Baptista et al., 2013). Hand and arm bimanual intensive therapy including lower extremities (HABIT-ILE) induced functional changes that were linked to an increase of DTI-based FA of the CST fibers [Bleyenheuft et al., under review]. It is unknown whether such changes could be obtained in adults with a chronic stroke and whether the amount of changes could match that of children.

In stroke patients, to date, very few intensive therapies have been thoroughly tested. These pioneering therapeutic experiments demonstrated that training needs to be intensive, high-dose, high-intensity, sustained, with carefully dosed feedback and reward, with clear goals with a meaningful significance for the patient (i.e. functional goals), based both on repetition (drill) and versatile exploration due to variable practice that fosters attention, "fun" and motivating, and based on the neurophysiological principles of motor learning. The most studied intensive therapy is the constraint-induced movement therapy (CIMT) (Hatem et al., 2016;

Pollock et al., 2015). This intensive intervention focuses on the paretic upper limb (UL) solely and is organized as blocks of training using motor learning concepts (i.e. practice specificity, context of learning, feedback, speed–accuracy trade-off, transfer of learning, etc.), aiming to elicit practice-induced brain changes arising from repetition, increasing movement complexity, motivation and reward. Motor learning plays a key role in post-stroke recovery (Krakauer, 2015). The refinement of the current models of motor learning/control recovery opens new perspectives in developing a new science of neurorehabilitation. Through training for instance, motor skill learning (MskL) leads to the acquisition and lasting retention of new sensorimotor aptitudes, i.e. "skills", characterized by enduring changes in the operating characteristics of motor patterns such as a shift in the speed/accuracy trade-off (SAT) and generalization (Haith & Krakauer, 2013; Shmuelof & Krakauer, 2011). MskL is a form of model-free learning driven by the reinforcement of successful actions (trial and error) based on reward-prediction error; it relies on several brain structures (Haith & Krakauer, 2013; Shmuelof & Krakauer, 2011). Furthermore, although efficient, CIMT does not involve the lower extremity of the patients, which can be impaired after stroke, as well as the rehabilitation of bimanual tasks and tasks requiring a combined use of upper and lower extremities. The impact of a unilateral stroke on bimanual motor control and ADL is so critical that it had led to the elaboration of specific scales quantifying these activity limitations such as the adult AHA (ad-AHA) (Van Gils et al., 2018) and ABILHAND scale (Penta et al., 2001), and that of neurorehabilitation programs tailoring bimanual activities (Luft et al., 2004; McCombe Waller & Whitall, 2004).

The aim of the present Grand Challenge is therefore to investigate the functional effectiveness of HABIT-ILE in chronic (>6 months) adult hemiparetic stroke patients while concurrently assessing the neuroplastic changes induced in their motor WM tracts during this training and relate them to perspectives of large motor improvements in adults with stroke, even in a chronic phase.

Research hypotheses of Grand Challenge 3

The goal of Grand Challenge 3 is to test the following hypotheses. In chronic hemiparetic adult stroke patients, compared to conventional rehabilitation, the HABIT-ILE intervention induces:

1. superior, clinically meaningful, gains on both activity limitation (1^{ary} clinical outcome) and other clinical outcomes in the three domains of the ICF (2^{ary} clinical outcomes) **(HP3.0)**
2. changes in white matter microstructure (WM-µS) integrity of the CST and cortico-cortical connections (1^{ary} imaging outcomes) **(HP3.1)**
3. changes in WM-µS integrity that correlate with mid-term changes in clinical outcomes (follow-up) **(HP3.2)**
4. functional gains predicted by baseline WM-µS integrity of the CST **(HP3.3)**
5. functional gains predicted by baseline aptitude to achieve bimanual motor skill learning (MSkL) **(HP3.4)**

We will systematically explore whether, in chronic hemiparetic stroke patients, the HABIT-ILE intervention induces superior, clinically meaningful, gains compared to conventional rehabilitation (**HP3.0**). The core experimental design of this cross-over randomized clinical trial (RCT) will be identical to that of Grand Challenge 2. *Of crucial importance will be the comparison between patient's subgroups training either with HABIT-ILE or with in-time-equivalent control period including daily activities and usual rehabilitation. Importantly, two common behavioral assessments will be used in CP children and adult stroke patients: AHA and COPM.*

Applying ICTEAM mathematical models, we will compute WM-µS integrity tools to test whether WM-µS integrity and lesions to specific brain structures impact functional changes (**HP3.1**). Using these ICTEAM advanced mathematical models, the WM-µS integrity of the CST (the key descending motor tract) and that of the whole brain will be explored with an unprecedented level of precision and compared to "classical" approaches using tractography with DTI measures as FA, RD and MD. Both the behavioral and MRI acquisitions will be performed pre- and post-intervention/control period and repeated at follow up (3 months), to explore

whether WM-µS integrity evolution correlates with mid-term clinical outcomes and to help refine ICTEAM models (**HP3.2**).

Given the limited resources in the "real-world" clinical environment, it is of paramount importance to establish biomarkers that can help predict which patient will benefit from specific therapies and thus to tailor rehabilitation. We will to explore whether baseline WM-µS integrity (**HP3.3**) and aptitude to achieve bimanual MskL on a rehabilitation robot (REAplan®, AXINESIS) (**HP3.4**) predict functional gains induced by the intensive HABIT-ILE therapy. Furthermore, the predictive model could be refined by combining the three aspects (clinical assessments, imaging and robotics).

METHODS AND STUDY DESIGN

In order to compare HABIT-ILE with the recovery of motor control during a time equivalent control period involving standard activity and conventional rehabilitation, we will run a single-blind randomized clinical trial (RCT) similar to Grand Challenge 2 with a parallel, cross-over group design (see Figure 5). The stroke patients will be randomly assigned either to a HABIT-ILE therapy for 10 days (5 days a week, 5 hours per day during 2 weeks; total = 50 hours) or to the time-equivalent control period with a minimization software to stratify patients for plegia/paresis/no motor deficit, gender, age, stroke in the (non-)dominant hemisphere, etc. The control group will follow its usual rehabilitation care and document all activities and therapeutic interventions during the initial 2-week period. A complete assessment will be performed for both groups before (T0) and after (T0+15d) the intervention/control period as well as 3 months post-intervention (T0+90d). An additional assessment will be added for the control group at the end of their delayed HABIT-ILE intervention, allowing us to collect data reflective of the effect of HABIT-ILE from a larger group. Each assessment will comprise clinical assessments, robotic measurements and neuroimaging data including multi-shell HARDI DW-MRI to be analyzed using ICTEAM frameworks for the extraction of WM µS indices.

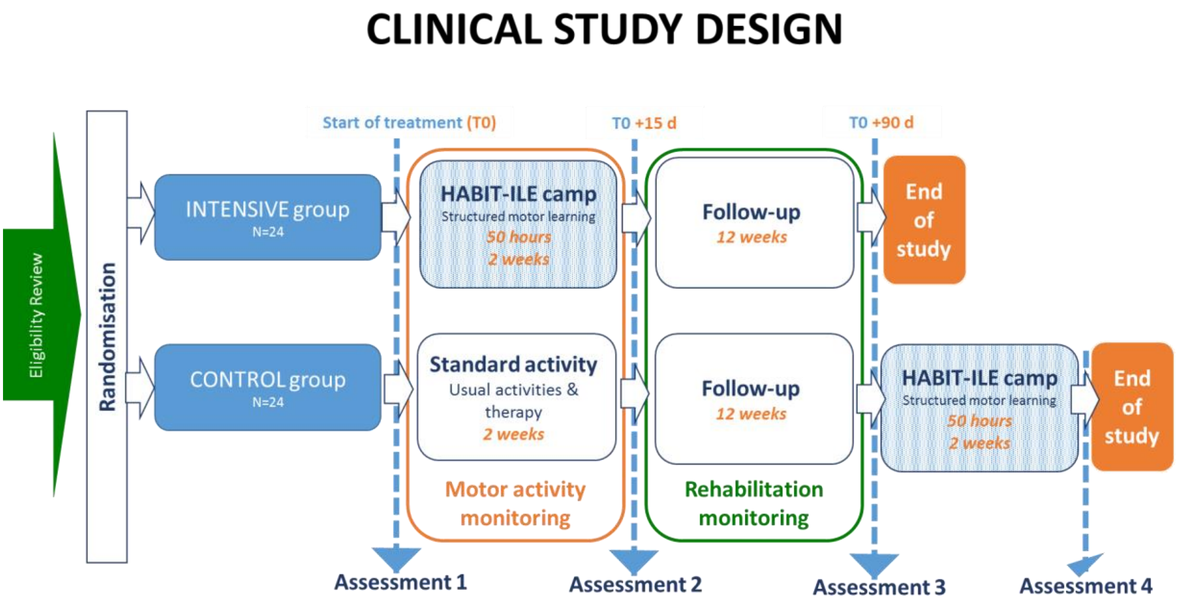

Figure 5: design of the randomized controled trial for a population of chronic stroke patients.

Population: N=48 (see sample size calculation). *Inclusion crit.:* being 1) a hemiparetic patient with a chronic stroke (>6 months), 2) 40-90 years old. *Exclusion crit.:* 1) alcohol/drug abuse, 2) pregnancy, 3) inability to understand/execute command, 4) major cognitive impairment interfering with the study (severe aphasia, psychiatric conditions), 5) uncontrolled health issues (cardiac/renal failure), 6) contraindications to MRI. In

order to span the largest possible range of neurological deficits, stroke of any type-size-location, involving either the dominant/non-dominant hemisphere will be included.

Procedures/Methodologies

The intensive motor skill training consists of an adaptation of HABIT-ILE. This intervention, designed initially for children with CP over 6 years old, is carried out in a camp-setting, with structured tasks of increasing motor difficulty and functional activities that require the use of both hands whilst sustaining postural activity or activities of the lower extremities (Bleyenheuft & Gordon, 2014). The therapy uses functional activities that are chosen according to individualized functional goals defined with the patients (e.g. drinking or dressing autonomously, cooking, etc.).

Although the basic principles of HABIT-ILE will be followed, the tasks and activities will be adapted to adults. The duration of activities as well as lower extremities/postural associations will also be adapted. Ten to twelve patients will undergo rehabilitation during one intensive session of 2 weeks; each patients having a full-time therapist dedicated to his/her treatment. The follow-up of the guidelines and the fidelity to the treatment principles will be ensured through the supervision of all interventions by the post-doc coordinator dedicated to the project and the expert team of Prof Bleyenheuft (MSLIN lab). As 50 to 60 hours of intensive therapy are needed to induce long-term motor changes with CIMT, this innovative project includes 5 hours per day of therapy with the same therapist for a total of 50 hours over 2 weeks.

As depicted in Figure 5, the time-equivalent control period consists of 2 weeks of the patient's normal life, with documentation (notebook) of all activities performed by the patient including his/her conventional therapy. After follow-up assessment (3 months) this group will undergo a delayed HABIT-ILE training and final assessment.

Evaluation criteria

Primary imaging outcomes (CST integrity measured with DTI, µS and myelination maps) will be measured at T0, T0+15 days and T0+90 days. We will use the classical DTI indicators of FA (index of fiber coherence and integrity), radial diffusivity (index of axon diameter and myelination), axial diffusivity, and mean diffusivity. These measures have been successfully used to demonstrate structural modifications of the CST, and provide an estimation of the fiber quality (Krägeloh-Mann & Cans, 2009). Crucially, we will use new advanced mathematical models which provide the opportunity to separately characterize fascicles of axons in areas of the brain where multiple WM tracts intersect, allowing thus to better identify fibers of the CST from other crossing tracts, quantify CST projections, as well as axonal loss (Scheck et al., 2015). Tractography will be used to highlight potential changes in CST organization (Bleyenheuft, Dricot, et al., 2015). Myelin maps will be used to assess global changes in myelination (Ganzetti et al., 2014). In addition, morphometric analysis (cortical thickness, cortical folding and sulcal depth) will be used to investigate potential changes in gray matter (Remer et al., 2017). Resting state fMRI will also be used to explore the potential changes in brain's functional organization and connectivity (Wen et al., 2019). The MRI data will be processed and analyzed under the combined supervision of Dr.Ir. L. Dricot (full-time fMRI expert at IoNS) and the ICTEAM team.

Clinical outcomes will investigate the **three domains of the ICF with** validated tests (Stucki et al., 2007). **At the body function and structure level, UL motor impairments** will be evaluated with validated scales: Fugl Meyer (FMA-UL), Box & Block Test and grip force. In addition a cognitive and visuospatial assessment will performed, including validated tests: GRECogVASC neuropsychological battery (French adaptation of the Harmonization Standards battery), Montreal Cognitive Assess (MoCA), Bell's Test, Corsi Blocks, movement control will be assessed through the Bimanual MsKL with robotics (see detailed description hereunder). **Activity limitations** will be evaluated with the modified Rankin Scale (mRS), Wolf Motor Function Test (SWMFT), Abilhand & Activlim questionnaires, the adult Assisting Hand Assessment (ad-AHA) (Krumlinde-Sundholm et al., 2019; Van Gils et al., 2018), and the Six-Minutes-Walking Test. **Social participation restriction** will be evaluated using the Stroke Impact Scale (SIS). The definition of functional goals will be performed using the Canadian Occupational Performance Measure (COPM) (Dedding et al., 2004).

The primary clinical outcome will be the ad-AHA. The other tests spanning the 3 ICF domains will be used as secondary clinical outcomes.

All physical assessments will be videotaped and blindly scored. All MRI data will be analyzed by an MRI specialist unaware of the treatment allocation.

Biomechanical outcome (bimanual MSkL on a robot): We implemented our serious game bimanual-CIRCUIT (MskL with a SAT (Doost et al., 2019; Yeganeh Doost et al., 2017)) in the new <u>bimanual</u> REAplan® environment. The subjects will use the robotic handles to move a common pointer as fast and accurately as possible along a complex circuit displayed on the immersive monitor, as illustrated in Figure 6. On-line (during training) & off-line (between training sessions) gains, retention and the learning curves will be quantified with velocity, accuracy, movement smoothness, SAT[1,4,5], and dynamics. The subjects will train during three days. During D3 session, generalization (another hallmark of MskL) will be quantified with another version of the CIRCUIT task [3,7,19]. <u>**Bimanual MskL**</u> is defined as the *retention* of SAT gains and/or as the generalization of SAT gains to an untrained version of the bimanual CIRCUIT.

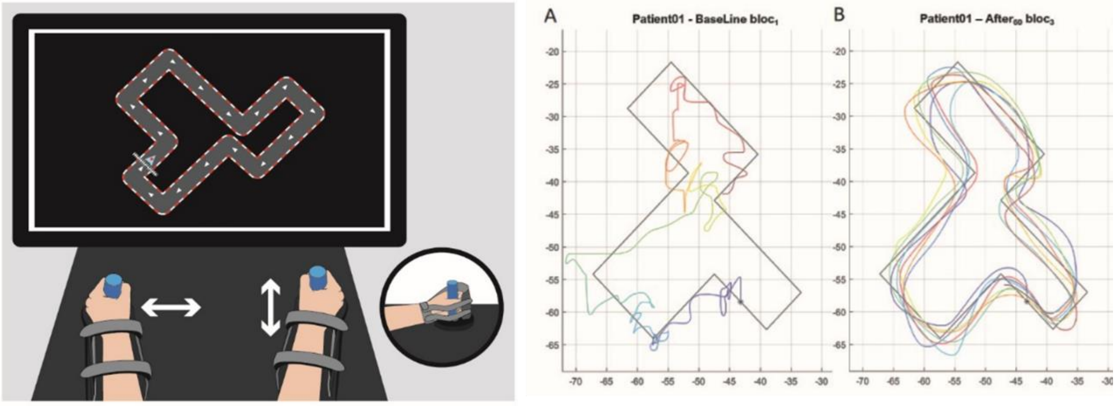

*Figure 6: The bimanual version of the **REAplan® robot** (AXINESIS / LOUVAIN BIONICS) (**left**) can mobilize both arms of the subject individually and record their positions (kinematics); it can also measure and interpret the forces exchange with the subject (dynamics). For the bimanual version of the serious game named CIRCUIT [46], the subjects will learn to coordinate bimanual movements using both robotic handles to displace a common cursor as fast and accurately as possible along a complex circuit displayed on the LCD monitor, completing as many laps as possible (speed constraint) in 1 minute while keeping the cursor within the track (accuracy constraint). **Right:** Bimanual MSkL in a chronic hemiparetic stroke patient (improvement pre-/post-training).*

Bimanual MskL will be quantified by **change in SAT** [28] [32] [33]. Error & velocity will be combined in a Performance Index (*PI = [velocity * 1/error] * C]*); error being the surface area between the pointer's trajectory and the midline of the track; and C a constant term used to balance the magnitudes of velocity and error. The PI is designed to increase when error diminishes and/or when velocity increases. Increase in PI reflects enhanced SAT, i.e. more skilled motor performance. **Generalization** will be quantified as "raw" PI_{GENER} / $PI_{LAST\_BLOCKS\_D3}$ for direct comparison. Changes in PI will be expressed as $(PI_{(x)} / PI_{BASE})$ allowing for log-transform and modelling. Based on pilot HI data, the PI of patients will be expressed as Z-scores and a cut-off (e.g. 2SD) will be derived to identity "non-learners", who will also be modeled separately in analyses.

STATISTICAL ANALYSIS

Since HABIT-ILE has never been applied in adult stroke patients, we based our sample size on motor changes observed previously in in school-aged children with brain lesions. We performed a sample size calculation based on the UL changes observed in the assisting hand assessment (AHA) [ref Bleyenheuft et al, 2015]; we expect a similar effect size here. Thus, our hypothesis is an incremental improvement of 1 SD between the HABIT-ILE group and the control group (between group difference=6, SD=5). With α=0.05 and a 1-β=0.9, a sample size of 16 participants per group is required. Considering potential drop-outs and data loss in MRI, **24 participants will be included in each group**. Neuroplastic changes and secondary outcomes will be compared

between groups using analysis of covariance (ANCOVA) to adjust for baseline measurement. Non-parametric methods will be performed whenever ANCOVA assumptions are not met.

The *R* software will be used under the supervision of statisticians for pre-specified stat. analyses as we did before [46,47, 57]. Separated generalized linear models (GLM) will be estimated for the CST and whole-brain WM (ICTEAM models) to infer the correlations between µS integrity at baseline/after intervention and the MSkL outcomes (bimanual SAT). Finally, an overall GLM will be estimated to combine CST and whole-brain µS integrity. The *R* software will also be used for bimanual MskL curves modelling.

PATIENT RECRUITMENT

We will recruit patients through regional and national stroke networks. For many years, patients have been recruited through the national Belgian Stroke Council (BSC) network. Yves Vandermeeren is the former President of the BSC Scientific Board, and an active member of the Scientific Board.

GRAND CHALLENGE 4: EXPLORING MECHANISMS OF TRAINING-INDUCED FUNCTIONAL RECOVERY THROUGH WM MICROSTRUCTURE IN VERY EARLY LESIONS VS ADULT ACQUIRED LESIONS

The identical experimental settings of WP 2 and 3 make it possible to directly compare WM baseline damage and training-induced changes between hemiplegic CP infants and adult stroke patients, both at the macroscopic level using whole-brain tractography and at the microscopic level using voxel-wise, model-based microstructural features. The deep underpinning of the differential repair mechanisms based on the initial conditions and age of the patients is likely to bring neurophysiological responses to the conditions allowing WM repair or training-induced changes. Investigating the best responders depending on initial conditions should help to better tailor treatment to each patient.

RESEARCH HYPOTHESES OF GRAND CHALLENGE 4

Grand Challenge 4 will test the following hypotheses**:**

1. Experience-induced remodeling of the damaged CST occurs both in CP infants and adult stroke patients undergoing HABIT-ILE, but is stronger in developing CP infants **(HP4.1)**;
2. Neuroinflammation quantified by DW-MRI biomarkers is more predominant in developing CP infants compared to chronic adult stroke patients **(HP4.2)**.

In both adult stroke patients and CP infants,

3. response to HABIT-ILE correlates *positively* with baseline DW-MRI markers quantifying the residual integrity of the damaged CST **(HP4.3)**;
4. response to HABIT-ILE correlates *negatively* with baseline DW-MRI markers quantifying a baseline enhancement of the ipsilateral, uncrossed CST **(HP4.4)**;
5. Combining DW-MRI µS markers of the CST with a whole-brain index of WM µS integrity correlates more closely with clinical response to HABIT-ILE than using exclusively the DW-MRI markers of the CST **(HP4.5).**

In a breakthrough exploratory step, we will try to combine functional and imaging data of CP infants and adult stroke patients using advanced model programming. Recovery in CP infants and stroke adults will be summarized and a normalization of the imaging data will be attempted for comparison in a common template. Whole-brain mapping of the two populations onto a common space may turn out to be too arduous due to very different brain sizes and large deformities in infants with CP. In that case, DW-MRI markers can always be compared after an adequate averaging over segmented sub-regions of the WM, such as the damaged or undamaged CST or regions not directly related to motor functions. Segmentation of the WM in the adult brain has become a routine task supported by many open-source software platforms. Improvements in the segmentation of the infant brain have also been gradually added to that existing software as well.

First, we hypothesize that, compared to baseline, HABIT-ILE intensive therapy induces remodeling of the µS indices of the damaged CST both in CP infants and adult stroke patients, but more so in developing CP infants **(HP4.1)**. The advantage of those DW-MRI indices is that their scale does not depend on brain size, brain age and are in theory only related to the morphology of the white matter independent of the imaging voxel size. If the hypothesis is verified (larger remodeling of the µS indexes in CP infants than in adult stroke patients), this would confirm potentially larger neuroplastic changes in the developing brain than in the aged brain, especially when combined with intensive rehabilitation, allowing for a lifespan quantification of neuroplastic potential. If the hypothesis is not verified, this would challenge the "classic" theory of larger neuroplastic potential in the developing brain.

Second, along the same line, we hypothesize that neuroinflammation quantified by DW-MRI biomarkers such as DIAMOND's extra-axonal restricted diffusivity is more persistent in developing CP infants than in chronic

adult stroke patients **(HP4.2)**. Indeed, a recent study in children with unilateral CP demonstrated that in contrast to typically developing children, children with unilateral CP do not present a maturational process of increasing CST integrity(Papadelis et al., 2019). The authors of this study also reported a decreased FA in both CSTs of children with unilateral CP. They attributed the absence of increase with age to a possible perinatal disruption of CST myelination and axonal integrity, due to persisting neuroinflammation during development. Stroke triggers a robust inflammatory response and, through multiple mechanisms such a glial activation, global brain inflammation might persistently shape the pathophysiology of brain injury after a stroke and promote decline of global brain functions like cognition, affecting the patients' long-term neurological outcome (Shi et al., 2019). Thus, in adults with stroke, neuroinflammation is major during the early (sub)acute phase of stroke and plays a key role in neuroplasticity (that could be both positive and/or deleterious) and, whereas neuroinflammation has been observed to decrease afterwards, it could persist in chronic stroke (Jayaraj et al., 2019; Shi et al., 2019; Thomas Carmichael, 2016).

Third, in an attempt to develop DW-MRI biomarkers common to CP infants and adult stroke patients, we will explore whether response to HABIT-ILE correlates *positively* with baseline DW-MRI markers quantifying the residual integrity of the damaged CST (**HP4.3**). We will determine which common level of residual integrity of the damaged CST predicts the larger functional gains after HABIT-ILE intensive therapy, and whether some threshold of damage limits response to HABIT-ILE. Potentially, based on ICTEAM algorithms, it would be possible to stratify the CP and stroke subjects in "good responders"", "poor responders" and "intermediate responders" and to extract baseline DW-MRI markers quantifying the residual integrity of the damaged CST for each subgroup. Alternatively, if CST integrity thresholds common to CP infants and adult stroke patients cannot be established, this would suggest again a different neuroplastic potential in the developing and aged brain.

Fourth, we hypothesize that response to HABIT-ILE correlates *negatively* with baseline DW-MRI markers quantifying a baseline enhancement of the ipsilateral, uncrossed CST (**HP4.4**), to a larger extent in CP infants than in adult stroke patients. The same procedures as for hypothesis **HP4.3** will be applied focusing on a negative correlation with the ipsilateral, uncrossed CST, expected to be stronger in CP infants. Indeed, it is expected to play a minor role, if any, in the damaged brain of adult primates (Zaaimi et al., 2012). In contrast, following early brain damage in CP, direct uncrossed axons can be preserved between the injured hemicortex and the more affected hemibody, which can thus be controlled by both sides of the brain [73]. The ipsilateral CST can therefore convey part of or all information for controlling the paretic hemibody (Guzzetta et al., 2007b). Studying carefully the relation between ipsilateral CST and response to HABIT-ILE will help further understand the mechanisms of recovery depending on initial condition, and *in fine* help fine-tune rehabilitation content regarding the initial role of the ipsilateral hemicortex and CST.

Fifth, combining DW-MRI markers of the CST with a whole-brain index of WM µS integrity may better predict the clinical response to HABIT-ILE than using the CST-specific markers alone (**HP4.5**). Multiple regression models will be tested to establish correlations between the clinical response to HABIT-ILE and µS indices of (1) the CST, (2) the corpus callosum and (3) whole-brain indices of WM µS integrity. Indeed, whereas a minimal level of CST integrity is undoubtedly needed to allow meaningful recovery, it becomes clear that cognitive components also play a key role in motor function recovery, at least in adult stroke patients (Rinne et al., 2018b). Exploring WM µS integrity beyond the CST may thus help in refining the stratification of patients in "good responders"", "poor responders" and "intermediate responders".

MICROMOTO: A HIGH RISK/HIGH GAIN PROJECT

The main risks are identified as

1) Damaged infant brains that present huge deformities might not be possibly aligned to systematic anatomical landmarks and atlases. For instance, programs as advanced as FreeSurfer still sometimes

fail to provide a reliable segmentation of the white and grey matter in brains due to the large deformities induced by the lesion. This may make it difficult to automate the detection of specific parts of the cortex (e.g., pre-motor, motor) and to cluster white matter streamlines into anatomically-identified tracts such as the cortico-spinal tract.

2) Most of the microstructural models in the literature, thus including those proposed in this project, have been validated for biophysical specificity (i.e., whether they non-invasively reveal what an invasive microscope analysis would show) in *animal* models using DW-MRI data acquired on preclinical scanners. The mathematical models have been applied to human data too but validation in those cases has been more empirical and generally less rigorous, du to the difficulty of performing histological studies. The main difference between preclinical and clinical MRI scanners, from a microstructure point of view, is the maximal strength of the diffusion gradients. The lower gradient intensities achievable with clinical scanners are known to impair our ability to measure the diameters of axons [Dyrby]. This is known as a resolution limit [Nilsson], whereby all diameters below about 3 microns (making up most of the brain axons) “look identical”, i.e., their signal contributions are the same and they cannot be distinguished.
3) Images acquisition in infants without sedation remains *per se* a huge challenge (no anesthesia in this project due to the risks associated with multiple anesthesia for young brains). Worldwide only a few teams have been able to develop processes allowing to acquire high-quality data from infants in spontaneous sleep. Although Y. Bleyenheuft’s team has recently developed a procedure and managed to acquire MRI data in 15 young children and infants, this is a major challenge in the study, especially regarding the time needed to acquire high-quality data, required for *Microstructure Fingerprinting.*
4) The HABIT-ILE intervention *per se* will require the participation and motivation of participants for many hours per day. This represents a potential high risk of either not meeting the high-intensity goals set in the HABIT-ILE therapy (e.g. due to lack of motivation leading to a small amount of repetitions) or of complete drop-out, both in infants and in adults who will mainly be up to 70 years of age.

Circumventing those limitations may be done via the following:

1) While cortical segmentation and white matter streamlines clustering should proceed fairly seamlessly in adult stroke patients, those tasks may prove more arduous in infant brains. In the worst case, segmentation will need to be performed manually based on anatomical landmarks for all 48 subjects of the CP study. However, the most likely scenario is a semi-automated framework where seeds would need to be manually placed at sites visually located on the MR images and automatic tractography would then be able to reconstruct tracts. In that way, tracts descending from specific cortical areas or the cortico-spinal tracts should be possible to reconstruct. Considering baseline biomarkers computed as average over the whole brain or over entire WM tracts is also a way to bypass the difficulty of bringing infant and adult brain into a common anatomical space.
2) The so-called resolution limit for axon diameters is a fundamental limitation of present-day DW-MRI technology, irrespective of the mathematical model used. However, the new GE scanner installed at Saint-Luc will deliver gradients up to 80 mT/m, the highest for any clinical system and a major improvement from the previous generation of MRI scanners, typically limited to 40-60 MT/m. Studies of sensitivity to microstructural features based on hardware constraints have been performed before and will be repeated during the preparation of DW-MRI sequences for this study, in collaboration with L. Dricot and the MRI physicist appointed by the MRI manufacturer [SPIE16]. Those studies will clearly delineate the uncertainty on our estimates of axon diameters. Of note is that our Microstructure Fingerprinting approach was based on the most accurate modeling approach available and was specifically designed to enhance sensitivity to axon diameter. Other microstructural features of importance such as axonal orientation and axonal density, expected to play a crucial role in the reorganization of the CST during neurorehabilitation, are much less affected by gradient strength and do not suffer from the transition to clinical settings. Finally, predictive biomarkers of motor recovery

can be found even if a bias persists between the biological grountruth and the DW-MRI –based µS estimates.

3) We will perform night MRIs with less noisy machines, shorter scanning sequences (possible thanks to a new equipment in our University Hopital Saint-Luc, Woluwe), very careful routines for gradual adaptation, specific installation and material for the infants. Repetitive MRIs under such conditions are successful in at least 70% of the infants[109].
4) We will provide time for participant before training to meet the team and discover the environment. Special attention will be paid in adults to the accessibility of the treatment room and the moment of the year the training will be performed (avoiding risks of contamination between fragile patients). Special attention will be paid for infants to the bonding with one therapist and providing routines during the day. Highly stimulating toys presented at the limit of reaching in the direction of movement expected and positive reinforcement will be used. These ingredients were successful in our pilot study including 4 children aged 12 to 18 months.

High gains:

If the hypotheses of improvement in WM patterns are met in this study high gains will be

1) Understanding the mechanisms of WM repair and thus better tailor neurorehabilitation programs
2) initiating a radical shift from the paradigms of rehabilitation currently applied (e.g. 2 time 30 minutes a week) to far more intensive programs
3) Huge changes for patients expectations: early improvements could highly decreased the lifelong disability of children, impacting their activity level, and their autonomy. In adults, if changes can be induced where our current healthcare systems considers these patients are not likely to recover anymore, the perspectives in term of autonomous life could be totally different.
4) Finally a high gain might be expected at the level of the society since the increased autonomy induced by such processes will also induce a decreased burden for the family of the patients and largely should allow more of these patients to play an active role in the society.

PART 2: PROJECT BUDGET AND MANAGEMENT

MICROMOTO BUDGET (5 years)

Personnel costs

The budget plan is based on the funding of

- 1 Post-doc coordinator of the whole ARC project during the first two years;
- 1 PhD student (Engineering- Signal Processing background) addressing HP1.1 to HP1.4, who will be based in ICTEAM and co-supervised by L. Dricot and B. Macq;
- 1 PhD student (Neurosciences background) addressing HP2.0 to HP2.4 based in IonS (CUSL) Woluwe under the supervision of Y. Bleyenheuft & Y. Vandermeeren, with support from L. Dricot;
- 1 PhD student (Neurosciences background) addressing HP3.0 to HP3.4 based in IonS CHU Mt Godinne & CUSL Woluwe under the supervision of Y. Vandermeeren & Y. Bleyenheuft, with support from L. Dricot.

The annual cost of Doctoral Grants at UCLouvain is EUR 34,122.12. Three PhD students for 4 years each amount to EUR 409,465.44. The annual cost of a post-doctoral coordinator is roughly EUR 85,000, over two years that is 85,000 *2=170,000. Total cost of staff is therefore **EUR 579.465**.

The 3 PhDs and the post-doc coordinator will each be granted a budget of EUR 12,000 for equipment and travels for the whole project duration (**total EUR 48,000**).

<u>Important notice:</u> We have restricted our budget to the funding of 3 PhD students and one post-doc fellow (during the first two years). We expect a multiplying factor of at least two of the size of our team as we will make each of our granted PhD students apply for a FRIA or FNRS grant. We will also complement our task force with several Master theses and internships.

<u>Operating costs (data acquisition)</u>

MRI costs (EUR 181.5/patient/assessment, i.e. EUR 150 + VAT): 358 assessments (48*4 assessments in children and 48*3 assessments in adults +24 additional assessments in the control group) = 360*181.5= EUR 65340.

Blind scoring of videotaped assessments (EUR 50/patient/outcome scored). AHA scored in 360 assessments (both children and adults), GMFM scored only in children for a total of 192 assessments. 552*50 = EUR 27,600 .

AXINESIS consulting for REAplan upgrades, LOUVAIN BIONICS software consulting: EUR 25,000.

Open Access publication of our scientific articles and (anonymized) data sets: EUR 15,000.

Rental costs of rehabilitation facilities: EUR 20,000.

Patients' travel costs between sites: EUR 20,000.

Total budget for operating costs is EUR **172,940.**

<u>Important notice:</u> the data acquisition budget and the related access to patients and equipment will give this project a considerable leading edge. The unique content of the generated dataset will be shared and exploited far beyond the specific goals of this project.

<u>Equipment costs</u>

Powerful computers for DW-MRI; behavioral data analyses and licenses as well as storage space and computing time at the Consortium des Équipements de Calcul Intensif (CÉCI) and the Center for High Performance Computing and Mass Storage (CISM): **EUR 25,000**.

<u>Other costs</u>

Travel for PI's and invited fellow researchers including M. Taquet (U of Oxford), J.-P. Thiran (EPFL), B. Scherrer (Harvard Med School), etc. : **EUR 20,000**.

<u>The total projected budget of the project is EUR 845,405.44.</u>

**Staff costs**				
Definition	**n**	**duration**	**cost/year/pers**	**Total cost**
Post-doc coordinator	1	2 years	85000	170000
PhD students	3	4 years	34122,12	409465,44
Total				579465,44
**Equipment, functioning and travel**	4	4 years	3000	48000
**Operating costs**	**n**		**cost/item**	**Total cost**
MRI costs	36O		150 + TVA=181.5	65340
Blind scoring of videotaped assessments (AHA children and adults / GMFM children)	552		50	27600
Consulting for REAplan upgrades				25000
building rental for intensive interventions				20000
Travel cost for patients from Mont-Godinne				20000
Open access publication and data sets				15000
Total				172940
**Equipment costs: powerful computer for DW-MRI/data analyses and licenses**				25000
**Travel for Pis and invited fellow researchers (Taquet, Thiran, Scherrer,…)**				**20000**
**Total budget**				**845405,44**

Management of the project

The Grand Challenges of this project each have a specific scientific background. Each of the research hypotheses will be explored and validated through specific studies described in the previous part of this document.

In order to maximize interactions inside the consortium, Dr Laurence Dricot (Ir, PhD) will be responsible to implement a global work plan overlaying the works inside the grand challenges and to coordinate DW-MRI data acquisition and analyses.

This workplan will be based on 5 work packages. Each of the researchers of the MicroMoto consortium will be committed to contributing to this overarching workplan. The timing of each workpackage is detailed in the Gantt chart.

WP1: administrative tasks

1. Recruitment of PhD students and post-doc
2. Ethical submission & approval
3. Insurance management
4. Trial registration in clinical trial database
5. Set-up & management of an online database
6. Patient recruitment management
7. Building rental management for intensive interventions / Recruitment of therapists in a 1:1 ratio
8. Assessments planning (MRI facilities / assessments rooms / availability of biomechanics material, availability & transport of participants).

WP2: set up of MRI condition and sequences

9. Developing DW-MRI sequences following pre-tests in a few patients
10. Testing MRI procedure in children < 18 months

WP3: Data acquisition

11. Running intensive rehabilitation processes
12. DW-MRI, functional and biomechanical data acquisition pre-post and 3 months after intensive process or control period
13. DW-MRI databases (existing data inside the group and publicly available, e.g. from the NIH).

WP4: Data Processing & analysis

14. Inventory of tools in use in IonS (MRIcron, Brain Voyager, FSL, Freesurfer etc.) and ICTEAM (3-D Slicer, Openreggui, etc.) for neuroimage processing
15. pipeline corrections DW-MRI images: denoising, Gibbs unringing, b0-field distortion, eddy current correction, registration of all images,...
16. estimation of diffusion models at ICTEAM
17. Repository of Matlab (or Python) code made available for the Challenges
18. Multimodal statistical processing

WP5: Clinical availability

19. Integration in vendors software platform for MRI
20. Workflow for patient data processing
21. Elaboration of an integrated framework available for clinic and research

The IoNS PI (YB & YV) and PhDs will constantly interact with the ICTEAM PI (BM) and PhD under the coordination of Dr.Ir. L. Dricot (full-time fMRI expert at IoNS) and the LOUVAIN BIONICS manager. Monthly meetings will be hosted by the LOUVAIN BIONICS consortium, to report WPs advances, task achieved to explore the HP, exchange multidisciplinary expertise (MD, engineer, IT expert…), elaborate solutions and coordinate the research efforts. For our multidisciplinary and translational MICROMOTO project, it is crucial to stimulate bilateral scientific exchanges between IoNS and ICTEAM researchers (fostered in the LOUVAIN BIONICS environment), so that expertise can be emulated, common language elaborated, and common goals refined.

Specific seminars will be organized yearly to present the research advances to colleagues from IoNS and LOUVAIN BIONICS and other UCLouvain experts in closely-related research fields. Dr. Ir. L. Dricot has regularly organized expert imaging teaching classes for MRI, DTI etc… She will keep inviting world-class experts in such boot-camps in order to provide up-to-date formation about brain MRI research to the MICROMOTO team.

Due to the multidisciplinary and translational nature of the MICROMOTO project, most of the scientific publications and international conferences will be shared by the MICROMOTO team; authorship will be shared and the contribution of each member will be determined accordingly to her/his direct input to the paper.

The software results and the data collected during the course of the project will be made public in compliance with the privacy rules (GDPR) and the legal framework laid out by the Louvain Technology Transfer Office.

The Gant chart p.29 shows the timing of the different workpackages over the 60 months of the ARC.

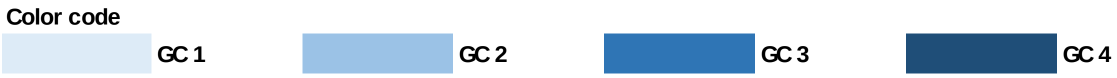

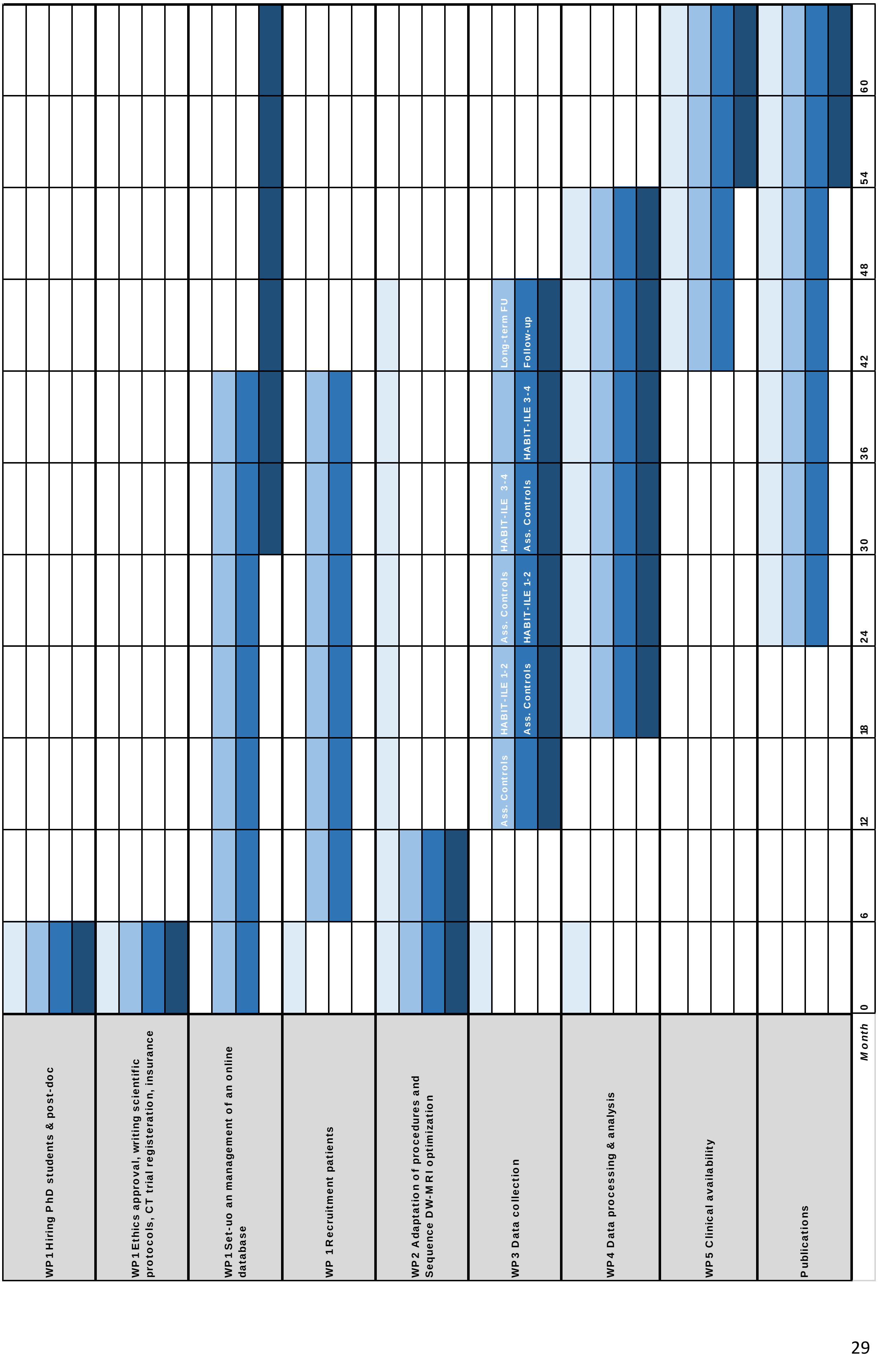

WP1 Hiring PhD students & post-doc
WP1 Ethics approval, writing scientific protocols, CT trial registeration, insurance
WP1 Set-uo an management of an online database
WP 1 Recruitment patients
WP2 Adaptation of procedures and Sequence DW-MRI optimization
WP3 Data collection
Ass. Controls
HABIT-ILE 1-2
Ass. Controls
Ass. Controls
HABIT-ILE 1-2
HABIT-ILE 3-4
Ass. Controls
HABIT-ILE 3-4
Long-term FU
Follow-up
WP4 Data processing & analysis
WP5 Clinical availability
Publications
Month
0
6
12
18
24
30
36
42
48
54
60

BIBLIOGRAPHY

Adluru, G., Gur, Y., Anderson, J. S., Richards, L. G., Adluru, N., & Dibella, E. V. R. (2014). Assessment of white matter microstructure in stroke patients using NODDI. *2014 36th Annual International Conference of the IEEE Engineering in Medicine and Biology Society, EMBC 2014*, 742–745. https://doi.org/10.1109/EMBC.2014.6943697

Alexander, D. C. (2008). A general framework for experiment design in diffusion MRI and its application in measuring direct tissue-microstructure features. *Magnetic Resonance in Medicine*, *60*(2), 439–448. https://doi.org/10.1002/mrm.21646

Basser, P. J., Mattiello, J., & Lebihan, D. (1994). Estimation of the Effective Self-Diffusion Tensor from the NMR Spin Echo. In *Journal of Magnetic Resonance, Series B* (Vol. 103, Issue 3, pp. 247–254). https://doi.org/10.1006/jmrb.1994.1037

Baud, O., Daire, J. L., Dalmaz, Y., Fontaine, R. H., Krueger, R. C., Sebag, G., Evrard, P., Gressens, P., & Verney, C. (2004). Gestational Hypoxia Induces White Matter Damage in Neonatal Rats: A New Model of Periventricular Leukomalacia. *Brain Pathology*, *14*(1), 1–10. https://doi.org/10.1111/j.1750-3639.2004.tb00492.x

Bleyenheuft, Y., Arnould, C., Brandao, M. B., Bleyenheuft, C., & Gordon, A. M. (2015). Hand and Arm Bimanual Intensive Therapy Including Lower Extremity (HABIT-ILE) in Children With Unilateral Spastic Cerebral Palsy: A Randomized Trial. *Neurorehabilitation and Neural Repair*, *29*(7), 645–657. https://doi.org/10.1177/1545968314562109

Bleyenheuft, Y., Dricot, L., Gilis, N., Kuo, H. C., Grandin, C., Bleyenheuft, C., Gordon, A. M., & Friel, K. M. (2015). Capturing neuroplastic changes after bimanual intensive rehabilitation in children with unilateral spastic cerebral palsy: A combined DTI, TMS and fMRI pilot study. *Research in Developmental Disabilities*, *43*, 136–149. https://doi.org/10.1016/j.ridd.2015.06.014

Bleyenheuft, Y., Ebner-Karestinos, D., Surana, B., Paradis, J., Sidiropoulos, A., Renders, A., Friel, K. M., Brandao, M., Rameckers, E., & Gordon, A. M. (2017). Intensive upper- and lower-extremity training for children with bilateral cerebral palsy: a quasi-randomized trial. *Developmental Medicine and Child Neurology*, *59*(6), 625–633. https://doi.org/10.1111/dmcn.13379

Bleyenheuft, Y., & Gordon, A. M. (2014). Hand-Arm Bimanual Intensive Therapy Including Lower Extremities (HABIT-ILE) for children with cerebral palsy. *Physical and Occupational Therapy in Pediatrics*, *34*(4), 390–403. https://doi.org/10.3109/01942638.2014.932884

Bleyenheuft, Y., Grandin, C. B., Cosnard, G., Olivier, E., & Thonnard, J. L. (2007). Corticospinal dysgenesis and upper-limb deficits in congenital hemiplegia: A diffusion tensor imaging study. *Pediatrics*, *120*(6), e1502-1511. https://doi.org/10.1542/peds.2007-0394

Bonita, R., & Beaglehole, R. (1988). Modification of Rankin Scale: Recovery of motor function after stroke. *Stroke; a Journal of Cerebral Circulation*, *19*, 1497–1500.

Brandão, M. B., Mancini, M. C., Ferre, C. L., Figueiredo, P. R. P., Oliveira, R. H. S., Gonçalves, S. C., Dias, M. C. S., & Gordon, A. M. (2018). Does Dosage Matter? A Pilot Study of Hand-Arm Bimanual Intensive Training (HABIT) Dose and Dosing Schedule in Children with Unilateral Cerebral Palsy. *Physical and Occupational Therapy in Pediatrics*, *38*(3), 227–242. https://doi.org/10.1080/01942638.2017.1407014

Byblow, W. D., Stinear, C. M., Barber, P. A., Petoe, M. A., & Ackerley, S. J. (2015). Proportional recovery after stroke depends on corticomotor integrity. *Annals of Neurology*, *78*(6), 848–859. https://doi.org/10.1002/ana.24472

Caby, B., Stamatakis, J., Laloux, P., Macq, B., & Vandermeeren, Y. (2011). Multi-modal movement reconstruction for stroke rehabilitation and performance assessment. *Journal on Multimodal User Interfaces*, *4*(3–4), 119–127. https://doi.org/10.1007/s12193-010-0056-y

Caverzasi, E., Papinutto, N., Castellano, A., Zhu, A. H., Scifo, P., Riva, M., Bello, L., Falini, A., Bharatha, A., & Henry, R. G. (2016). Neurite Orientation Dispersion and Density Imaging Color Maps to Characterize Brain Diffusion in Neurologic Disorders. *Journal of Neuroimaging*, *26*(5), 494–498. https://doi.org/10.1111/jon.12359

Dean, D. C., Dirks, H., O'Muircheartaigh, J., Walker, L., Jerskey, B. A., Lehman, K., Han, M., Waskiewicz, N., & Deoni, S. C. L. (2014). Pediatric neuroimaging using magnetic resonance imaging during non-sedated sleep. *Pediatric Radiology*, *44*(1), 64–72. https://doi.org/10.1007/S00247-013-2752-8

Dedding, C., Cardol, M., Eyssen, I. C. J. M., Dekker, J., & Beelen, A. (2004). Validity of the Canadian occupational performance measure: A client-centred outcome measurement. *Clinical Rehabilitation*, *18*(6), 660–667. https://doi.org/10.1191/0269215504cr746oa

Deluca, S. C., Case-Smith, J., Stevenson, R., & Ramey, S. L. (2012). Constraint-induced movement therapy (CIMT) for young children with cerebral palsy: Effects of therapeutic dosage. *Journal of Pediatric Rehabilitation Medicine*, *5*(2), 133–142. https://doi.org/10.3233/PRM-2012-0206

Deoni, S. C. L., Dean, D. C., Remer, J., Dirks, H., & O'Muircheartaigh, J. (2015). Cortical maturation and myelination in healthy toddlers and young children. *NeuroImage*, *115*, 147–161. https://doi.org/10.1016/j.neuroimage.2015.04.058

Dewilde, S., Annemans, L., Peeters, A., Hemelsoet, D., Vandermeeren, Y., Desfontaines, P., Brouns, R., Vanhooren, G., Cras, P., Michielsens, B., Redondo, P., & Thijs, V. (2017). Modified Rankin scale as a determinant of direct medical costs after stroke. *International Journal of Stroke*, *12*(4), 392–400. https://doi.org/10.1177/1747493017691984

Doost, M. Y., Orban de Xivry, J. J., Herman, B., Vanthournhout, L., Riga, A., Bihin, B., Jamart, J., Laloux, P., Raymackers, J. M., & Vandermeeren, Y. (2019). Learning a Bimanual Cooperative Skill in Chronic Stroke Under Noninvasive Brain Stimulation: A Randomized Controlled Trial. *Neurorehabilitation and Neural Repair*, *33*(6), 486–498. https://doi.org/10.1177/1545968319847963

Dunn, W., & Westman, K. (1997). The Sensory Profile: The Performance of a National Sample of Children Without Disabilities. *American Journal of Occupational Therapy*, *51*(1), 25–34. https://doi.org/10.5014/ajot.51.1.25

Eaton-Rosen, Z., Melbourne, A., Orasanu, E., Cardoso, M. J., Modat, M., Bainbridge, A., Kendall, G. S., Robertson, N. J., Marlow, N., & Ourselin, S. (2015). Longitudinal measurement of the developing grey matter in preterm subjects using multi-modal MRI. *NeuroImage*. https://doi.org/10.1016/j.neuroimage.2015.02.010

Eaton-Rosen, Z., Scherrer, B., Melbourne, A., Ourselin, S., Neil, J. J., & Warfield, S. K. (2017). Investigating the maturation of microstructure and radial orientation in the preterm human cortex with diffusion MRI. *NeuroImage*, *162*, 65–72. https://doi.org/10.1016/j.neuroimage.2017.08.013

Eliasson, A. C., Nordstrand, L., Ek, L., Lennartsson, F., Sjöstrand, L., Tedroff, K., & Krumlinde-Sundholm, L. (2018). The effectiveness of Baby-CIMT in infants younger than 12 months with clinical signs of unilateral-cerebral palsy; an explorative study with randomized design. *Research in Developmental Disabilities*, *72*, 191–201. https://doi.org/10.1016/j.ridd.2017.11.006

Eliasson, A. C., Shaw, K., Berg, E., & Krumlinde-Sundholm, L. (2011). An ecological approach of Constraint Induced Movement Therapy for 2-3-year-old children: A randomized control trial. *Research in Developmental Disabilities*, *32*(6), 2820–2828. https://doi.org/10.1016/j.ridd.2011.05.024

Eyre, J. A., Taylor, J. P., Villagra, F., Smith, M., & Miller, S. (2001). Evidence of activity-dependent withdrawal of corticospinal projections during human development. *Neurology*, *57*(9), 1543–1554. https://doi.org/10.1212/WNL.57.9.1543

Favrais, G., Van De Looij, Y., Fleiss, B., Ramanantsoa, N., Bonnin, P., Stoltenburg-Didinger, G., Lacaud, A., Saliba, E., Dammann, O., Gallego, J., Sizonenko, S., Hagberg, H., Lelièvre, V., & Gressens, P. (2011). Systemic inflammation disrupts the developmental program of white matter. *Annals of Neurology*, *70*(4), 550–565. https://doi.org/10.1002/ana.22489

Friel, K., Chakrabarty, S., Kuo, H. C., & Martin, J. (2012). Using motor behavior during an early critical period to restore skilled limb movement after damage to the corticospinal system during development. *Journal of Neuroscience*, *32*(27), 9265–9276. https://doi.org/10.1523/JNEUROSCI.1198-12.2012

Ganzetti, M., Wenderoth, N., & Mantini, D. (2014). Whole brain myelin mapping using T1- and T2-weighted MR imaging data. *Frontiers in Human Neuroscience*, *8*, 671. https://doi.org/10.3389/fnhum.2014.00671

Graham, H. K. (2016). Primer: Cerebral Palsy. *Nature Reviews/Disease Primers*, *2*(15082).

Greaves, S., Imms, C., Dodd, K., & Krumlinde-Sundholm, L. (2013). Development Of The Mini-Assisting Hand Assessment: Evidence For Content And Internal Scale Validity. *Developmental Medicine and Child Neurology*, *55*(11), 1030–1037. https://doi.org/10.1111/dmcn.12212

Guzzetta, A., Bonanni, P., Biagi, L., Tosetti, M., Montanaro, D., Guerrini, R., & Cioni, G. (2007a). Reorganisation of the somatosensory system after early brain damage. *Clinical Neurophysiology*, *118*(5), 1110–1121. https://doi.org/10.1016/j.clinph.2007.02.014

Guzzetta, A., Bonanni, P., Biagi, L., Tosetti, M., Montanaro, D., Guerrini, R., & Cioni, G. (2007b). Reorganisation of the somatosensory system after early brain damage. *Clin Neurophysiol*, *118*(5), 1110–1121. https://doi.org/10.1016/j.clinph.2007.02.014

Hackett, M. L., Duncan, J. R., Anderson, C. S., Broad, J. B., & Bonita, R. (2000). Health-related quality of life among long-term survivors of stroke: Results from the Auckland stroke study, 1991-1992. *Stroke*, *31*, 440–447. https://doi.org/10.1161/01.STR.31.2.440

Haith, A. M., & Krakauer, J. W. (2013). Model-based and model-free mechanisms of human motor learning. *Advances in Experimental Medicine and Biology*, *782*, 1–21. https://doi.org/10.1007/978-1-4614-5465-6_1

Hankey, G. J., Jamrozik, K., Broadhurst, R. J., Forbes, S., & Anderson, C. S. (2002). Long-term disability after first-ever stroke and related prognostic factors in the Perth Community Stroke Study, 1989-1990. *Stroke*, *33*, 1034–1040. https://doi.org/10.1161/01.STR.0000012515.66889.24

Hardwick, R. M., Rajan, V. A., Bastian, A. J., Krakauer, J. W., & Celnik, P. A. (2016). Motor Learning in Stroke: Trained Patients Are Not Equal to Untrained Patients With Less Impairment. *Neurorehabilitation and Neural Repair*, *31*, 178–189. https://doi.org/10.1177/1545968316675432

Hatem, S. M., Saussez, G., della Faille, M., Prist, V., Zhang, X., Dispa, D., & Bleyenheuft, Y. (2016). Rehabilitation of motor function after stroke: A multiple systematic review focused on techniques to stimulate upper extremity recovery. *Frontiers in Human Neuroscience*, *10*, 442. https://doi.org/10.3389/fnhum.2016.00442

Jayaraj, R. L., Azimullah, S., Beiram, R., Jalal, F. Y., & Rosenberg, G. A. (2019). Neuroinflammation: friend and foe for ischemic stroke. *Journal of Neuroinflammation*, *16*(1). https://doi.org/10.1186/S12974-019-1516-2

Jelescu, I. O., & Budde, M. D. (2017). Design and validation of diffusion MRI models of white matter. In *Frontiers in Physics* (Vol. 5, p. 61). https://doi.org/10.3389/fphy.2017.00061

Jelescu, I. O., Veraart, J., Fieremans, E., & Novikov, D. S. (2016). Degeneracy in model parameter estimation for multi-compartmental diffusion in neuronal tissue. *NMR in Biomedicine*, *29*(1), 33–47. https://doi.org/10.1002/nbm.3450

Jeurissen, B., Leemans, A., Tournier, J. D., Jones, D. K., & Sijbers, J. (2013). Investigating the prevalence of complex fiber configurations in white matter tissue with diffusion magnetic resonance imaging. *Human Brain Mapping*, *34*(11), 2747–2766. https://doi.org/10.1002/hbm.22099

Kantak, S., McGrath, R., & Zahedi, N. (2016). Goal conceptualization and symmetry of arm movements affect bimanual coordination in individuals after stroke. *Neuroscience Letters*, *626*, 86–93. https://doi.org/10.1016/j.neulet.2016.04.064

Khetani, M. A., Graham, J. E., Davies, P. L., Law, M. C., & Simeonsson, R. J. (2015). Psychometric properties of the young children's participation and environment measure. *Archives of Physical Medicine and Rehabilitation*, *96*(2), 307–316. https://doi.org/10.1016/j.apmr.2014.09.031

Krägeloh-Mann, I., & Cans, C. (2009). Cerebral palsy update. *Brain and Development*, *31*(7), 537–544. https://doi.org/10.1016/j.braindev.2009.03.009

Krakauer, J. W. (2015). The applicability of motor learning to neurorehabilitation. In *Oxford Textbook of Neurorehabilitation* (pp. 55–63). https://doi.org/10.1093/med/9780199673711.001.0001

Kramer, J. M., Liljenquist, K., & Coster, W. J. (2016). Validity, reliability, and usability of the Pediatric Evaluation of Disability Inventory-Computer Adaptive Test for autism spectrum disorders. *Developmental Medicine and Child Neurology*, *58*(3), 255–261. https://doi.org/10.1111/dmcn.12837

Krumlinde-Sundholm, L., Lindkvist, B., Plantin, J., & Hoare, B. (2019). Development of the assisting hand assessment for adults following stroke: a Rasch-built bimanual performance measure. *Disability and Rehabilitation*, *41*(4), 472–480. https://doi.org/10.1080/09638288.2017.1396365

Lampinen, B., Szczepankiewicz, F., Mårtensson, J., van Westen, D., Sundgren, P. C., & Nilsson, M. (2017). Neurite density imaging versus imaging of microscopic anisotropy in diffusion MRI: A model comparison using spherical tensor encoding. *NeuroImage*, *147*(July 2016), 517–531. https://doi.org/10.1016/j.neuroimage.2016.11.053

Li, Q., & Martin, J. H. (2000). Postnatal development of differential projections from the caudal and rostral motor cortex subregions. *Experimental Brain Research*, *134*(2), 187–198. https://doi.org/10.1007/s002210000454

Liu, M., Gross, D. W., Wheatley, B. M., Concha, L., & Beaulieu, C. (2013). The acute phase of Wallerian degeneration: Longitudinal diffusion tensor imaging of the fornix following temporal lobe surgery. *NeuroImage*, *74*, 128–139. https://doi.org/10.1016/j.neuroimage.2013.01.069

Luft, A. R., McCombe-Waller, S., Whitall, J., Forrester, L. W., Macko, R., Sorkin, J. D., Schulz, J. B., Goldberg, A. P., & Hanley, D. F. (2004). Repetitive bilateral arm training and motor cortex activation in chronic stroke: A randomized controlled trial. *Journal of the American Medical Association*, *292*(15), 1853–1861. https://doi.org/10.1001/jama.292.15.1853

Maes, C., Gooijers, J., Orban de Xivry, J. J., Swinnen, S. P., & Boisgontier, M. P. (2017). Two hands, one brain, and aging. *Neuroscience and Biobehavioral Reviews*, *75*, 234–256. https://doi.org/10.1016/j.neubiorev.2017.01.052

Martin, J. H. (2005). The corticospinal system: From development to motor control. *Neuroscientist*, *11*(2), 161–173. https://doi.org/10.1177/1073858404270843

Martin, J. H., & Lee, S. J. (1999). Activity-dependent competition between developing corticospinal terminations. *NeuroReport*, *10*(11), 2277–2282. https://doi.org/10.1097/00001756-199908020-00010

McCombe Waller, S., & Whitall, J. (2004). Fine motor control in adults with and without chronic hemiparesis: Baseline comparison to nondisabled adults and effects of bilateral arm training. *Archives of Physical Medicine and Rehabilitation*, *85*(7), 1076–1083. https://doi.org/10.1016/j.apmr.2003.10.020

McKenzie, I. A., Ohayon, D., Li, H., De Faria, J. P., Emery, B., Tohyama, K., & Richardson, W. D. (2014). Motor skill learning requires active central myelination. *Science*, *346*(6207), 318–322. https://doi.org/10.1126/science.1254960

Mozaffarian, D., Benjamin, E. J., Go, A. S., Arnett, D. K., Blaha, M. J., Cushman, M., Das, S. R., de Ferranti, S., Després, J.-P., Fullerton, H. J., Howard, V. J., Huffman, M. D., Isasi, C. R., Jiménez, M. C., Judd, S. E., Kissela, B. M., Lichtman, J. H., Lisabeth, L. D., Liu, S., … Turner, M. B. (2016). Heart Disease and Stroke Statistics—2016 Update. *Circulation*, *133*, e38-360. https://doi.org/10.1161/cir.0000000000000350

Nemanich, S. T., Mueller, B. A., & Gillick, B. T. (2019). Neurite orientation dispersion and density imaging quantifies corticospinal tract microstructural organization in children with unilateral cerebral palsy. *Human Brain Mapping*, *40*(17), 4888–4900. https://doi.org/10.1002/hbm.24744

Noirhomme, Q., Ferrant, M., Vandermeeren, Y., Olivier, E., Macq, B., & Cuisenaire, O. (2004). Registration and real-time visualization of transcranial magnetic stimulation with 3-D MR images. *IEEE Transactions on Biomedical Engineering*, *51*(11), 1994–2005. https://doi.org/10.1109/TBME.2004.834266

Nordstrand, L., Holmefur, M., Kits, A., & Eliasson, A. C. (2015). Improvements in bimanual hand function after baby-CIMT in two-year old children with unilateral cerebral palsy: A retrospective study. *Research in Developmental Disabilities*, *41*, 86–93. https://doi.org/10.1016/j.ridd.2015.05.003

novak, I., Mcintyre, S., Morgan, C., Campbell, L., Dark, L., Morton, N., Stumbles, E., Wilson, S. A., & Goldsmith, S. (2013). A systematic review of interventions for children with cerebral palsy: State of the evidence. *Developmental Medicine and Child Neurology*, *55*(10), 885–910. https://doi.org/10.1111/dmcn.12246

Papadelis, C., Kaye, H., Shore, B., Snyder, B., Grant, P. E., & Rotenberg, A. (2019). Maturation of Corticospinal Tracts in Children With Hemiplegic Cerebral Palsy Assessed by Diffusion Tensor Imaging and Transcranial Magnetic Stimulation. *Frontiers in Human Neuroscience*, *13*. https://doi.org/10.3389/FNHUM.2019.00254

Penta, M., Tesio, L., Arnould, C., Zancan, A., & Thonnard, J. L. (2001). The ABILHAND questionnaire as a measure of manual ability in chronic stroke patients: Rasch-based validation and relationship to upper limb impairment. *Stroke*, *32*(7), 1627–1634. https://doi.org/10.1161/01.STR.32.7.1627

Pollock, A., Farmer, S. E., Brady, M. C., Langhorne, P., Mead, G. E., Mehrholz, J., & Van Wijck, F. (2015). Cochrane overview: Interventions for improving upper limb function after stroke. *Stroke*, *11*. https://doi.org/10.1161/STROKEAHA.114.008295

Provost, B., & Oetter, P. (1994). The sensory rating scale for infants and Young children: Development and reliability. *Physical and Occupational Therapy in Pediatrics*, *13*(4), 15–35. https://doi.org/10.1080/J006v13n04_02

Ransburg, N., Reiser, M., Munzert, J., Jovanovic, B., & Schwarzer, G. (2017). Concurrent anticipation of two object dimensions during grasping in 10-month-old infants: A quantitative analysis. *Infant Behavior and Development*, *48*, 164–174. https://doi.org/10.1016/j.infbeh.2017.04.003

Remer, J., Croteau-Chonka, E., Dean, D. C., D'Arpino, S., Dirks, H., Whiley, D., & Deoni, S. C. L. (2017). Quantifying cortical development in typically developing toddlers and young children, 1–6 years of age. *NeuroImage*, *153*, 246–261. https://doi.org/10.1016/j.neuroimage.2017.04.010

Rensonnet, G., Scherrer, B., Girard, G., Jankovski, A., Warfield, S. K., Macq, B., Thiran, J. P., & Taquet, M. (2019). Towards microstructure fingerprinting: Estimation of tissue properties from a dictionary of Monte Carlo diffusion MRI simulations. *NeuroImage*, *184*(May 2018), 964–980. https://doi.org/10.1016/j.neuroimage.2018.09.076

Rinne, P., Hassan, M., Fernandes, C., Han, E., Hennessy, E., Waldman, A., Sharma, P., Soto, D., Leech, R., Malhotra, P. A., & Bentley, P. (2018a). Motor dexterity and strength depend upon integrity of the attention-control system. *Proceedings of the National Academy of Sciences of the United States of America*, *115*(3), E536–E545. https://doi.org/10.1073/pnas.1715617115

Rinne, P., Hassan, M., Fernandes, C., Han, E., Hennessy, E., Waldman, A., Sharma, P., Soto, D., Leech, R., Malhotra, P. A., & Bentley, P. (2018b). Motor dexterity and strength depend upon integrity of the attention-control system. *Proceedings of the National Academy of Sciences of the United States of America*, *115*(3), E536–E545. https://doi.org/10.1073/PNAS.1715617115

Rojas-Vite, G., Coronado-Leija, R., Narvaez-Delgado, O., Ramírez-Manzanares, A., Marroquín, J. L., Noguez-Imm, R., Aranda, M. L., Scherrer, B., Larriva-Sahd, J., & Concha, L. (2019). Histological validation of per-bundle water diffusion metrics within a region of fiber crossing following axonal degeneration. *NeuroImage*, *201*. https://doi.org/10.1016/j.neuroimage.2019.116013

Russell, D. J., Avery, L. M., Walter, S. D., Hanna, S. E., Bartlett, D. J., Rosenbaum, P. L., Palisano, R. J., & Gorter, J. W. (2010). Development and validation of item sets to improve efficiency of administration of the 66-item Gross Motor Function Measure in children with cerebral palsy. *Developmental Medicine and Child Neurology*, *52*(2), e48-54. https://doi.org/10.1111/j.1469-8749.2009.03481.x

Sakzewski, L., Provan, K., Ziviani, J., & Boyd, R. N. (2015). Comparison of dosage of intensive upper limb therapy for children with unilateral cerebral palsy: How big should the therapy pill be? *Research in Developmental Disabilities*, *37*, 9–16. https://doi.org/10.1016/j.ridd.2014.10.050

Sampaio-Baptista, C., Khrapitchev, A. A., Foxley, S., Schlagheck, T., Scholz, J., Jbabdi, S., DeLuca, G. C., Miller, K. L., Taylor, A., Thomas, N., Kleim, J., Sibson, N. R., Bannerman, D., & Johansen-Berg, H. (2013). Motor skill learning induces changes in white matter microstructure and myelination. *Journal of Neuroscience*, *33*(50), 19499–19503. https://doi.org/10.1523/JNEUROSCI.3048-13.2013

Scheck, S. M., Pannek, K., Raffelt, D. A., Fiori, S., Boyd, R. N., & Rose, S. E. (2015). Structural connectivity of the anterior cingulate in children with unilateral cerebral palsy due to white matter lesions. *NeuroImage: Clinical*, *9*, 498–505. https://doi.org/10.1016/j.nicl.2015.09.014

Scherrer, B., Schwartzman, A., Taquet, M., Sahin, M., Prabhu, S. P., & Warfield, S. K. (2016). Characterizing Brain Tissue by Assessment of the Distribution of Anisotropic Microstructural Environments in Diffusion-Compartment Imaging (DIAMOND). *Magnetic Resonance in Medicine*, *76*(3), 963–977. https://doi.org/10.1002/mrm.25912

Scherrer, B., Taquet, M., Schwartzman, A., St-Onge, E., Rensonnet, G., Prabhu, S. P., & Warfield, S. K. (2017). Decoupling axial and radial tissue heterogeneity in diffusion compartment imaging. In *Lecture Notes in Computer Science (including subseries Lecture Notes in Artificial Intelligence and Lecture Notes in Bioinformatics): Vol. 10265 LNCS*. https://doi.org/10.1007/978-3-319-59050-9_35

Scherrer, B., Taquet, M., & Warfield, S. K. (2013). Reliable selection of the number of fascicles in diffusion images by estimation of the generalization error. *Lecture Notes in Computer Science (Including Subseries Lecture Notes in Artificial Intelligence and Lecture Notes in Bioinformatics)*, *7917 LNCS*, 742–753. https://doi.org/10.1007/978-3-642-38868-2_62

Schilling, K., Gao, Y., Janve, V., Stepniewska, I., Landman, B. A., & Anderson, A. W. (2017). Can increased spatial resolution solve the crossing fiber problem for diffusion MRI? *NMR in Biomedicine*, *30*(12), 1–16. https://doi.org/10.1002/nbm.3787

Shi, K., Tian, D. C., Li, Z. G., Ducruet, A. F., Lawton, M. T., & Shi, F. D. (2019). Global brain inflammation in stroke. *The Lancet. Neurology*, *18*(11), 1058–1066. https://doi.org/10.1016/S1474-4422(19)30078-X

Shmuelof, L., & Krakauer, J. W. (2011). Are we ready for a natural history of motor learning? *Neuron*, *72*, 469–476. https://doi.org/10.1016/j.neuron.2011.10.017

Staudt, M. (2010). Brain Plasticity Following Early Life Brain Injury: Insights From Neuroimaging. *Seminars in Perinatology*, *34*(1), 87–92. https://doi.org/10.1053/j.semperi.2009.10.009

Stinear, C. (2010). Prediction of recovery of motor function after stroke. *The Lancet Neurology*, *9*, 1228–1232. https://doi.org/10.1016/S1474-4422(10)70247-7

Stucki, G., Cieza, A., & Melvin, J. (2007). The international classification of functioning, disability and health: A unifying model for the conceptual description of the rehabilitation strategy. *Journal of Rehabilitation Medicine*, *39*(4), 286–292. https://doi.org/10.2340/16501977-0041

Taquet, M., Jankovski, A., Rensonnet, G., Jacobs, D., des Rieux, A., Macq, B., Warfield, S. K., & Scherrer, B. (2019). Extra-axonal restricted diffusion as an in-vivo marker of reactive microglia. *Scientific Reports*, *9*(1), 1–10. https://doi.org/10.1038/s41598-019-50432-5

Taquet, M., Scherrer, B., Benjamin, C., Prabhu, S., MacQ, B., & Warfield, S. K. (2012). Interpolating multi-fiber models by Gaussian mixture simplification. *Proceedings - International Symposium on Biomedical Imaging*. https://doi.org/10.1109/ISBI.2012.6235708

Taquet, M., Scherrer, B., Commowick, O., Peters, J. M., Sahin, M., Macq, B., & Warfield, S. K. (2014). A mathematical framework for the registration and analysis of multi-fascicle models for population studies of the brain microstructure. *IEEE Transactions on Medical Imaging*, *33*(2), 504–517. https://doi.org/10.1109/TMI.2013.2289381

Taquet, M., Scherrer, B., Peters, J. M., Prabhu, S. P., & Warfield, S. K. (2014). A fully Bayesian inference framework for population studies of the brain microsctructure. *International Conference on Medical Image Computing and Computer-Assisted Intervention*, *8673 LNCS*(PART 1), 25–32. https://doi.org/10.1007/s11103-011-9767-z.Plastid

Taub, E., Ramey, S. L., DeLuca, S., & Echols, K. (2004). Efficacy of Constraint-Induced Movement Therapy for Children with Cerebral Palsy with Asymmetric Motor Impairment. *Pediatrics*, *113*(2), 305–312. https://doi.org/10.1542/peds.113.2.305

Thomas Carmichael, S. (2016). Emergent properties of neural repair: elemental biology to therapeutic concepts. *Annals of Neurology*, *79*(6), 895–906. https://doi.org/10.1002/ANA.24653

Tuch, D. S., Reese, T. G., Wiegell, M. R., Makris, N., Belliveau, J. W., & Van Wedeen, J. (2002). High angular resolution diffusion imaging reveals intravoxel white matter fiber heterogeneity. *Magnetic Resonance in Medicine*, *48*(4), 577–582. https://doi.org/10.1002/mrm.10268

Van Cauwenberghe, E., Gubbels, J., De Bourdeaudhuij, I., & Cardon, G. (2011). Feasibility and validity of accelerometer measurements to assess physical activity in toddlers. *International Journal of Behavioral Nutrition and Physical Activity*, *8*(1), 67. https://doi.org/10.1186/1479-5868-8-67

Van Gils, A., Meyer, S., Van Dijk, M., Thijs, L., Michielsen, M., Lafosse, C., Truyens, V., Oostra, K., Peeters, A., Thijs, V., Feys, H., Krumlinde-Sundholm, L., Kos, D., & Verheyden, G. (2018). The Adult Assisting Hand Assessment Stroke: Psychometric Properties of an Observation-Based Bimanual Upper Limb Performance Measurement. *Archives of Physical Medicine and Rehabilitation*, *99*(12), 2513–2522. https://doi.org/10.1016/j.apmr.2018.04.025

Van Hecke, W., Emsell, L., & Sunaert, S. (2016). Diffusion Tensor Imaging: A Practical Handbook. In *Diffusion Tensor Imaging: A Practical Handbook*. https://doi.org/10.1007/978-1-4939-3118-7

Van Panhuis, W. G., Grefenstette, J., Jung, S. Y., Chok, N. S., Cross, A., Eng, H., Lee, B. Y., Zadorozhny, V., Brown, S., Cummings, D., & Burke, D. S. (2013). Contagious diseases in the United States from 1888 to the present. *New England Journal of Medicine*, *369*(22), 2152. https://doi.org/10.1056/NEJMms1215400

Van Steenwinckel, J., Schang, A. L., Sigaut, S., Chhor, V., Degos, V., Hagberg, H., Baud, O., Fleiss, B., & Gressens, P. (2014). Brain damage of the preterm infant: New insights into the role of inflammation. *Biochemical Society Transactions*, 557–563. https://doi.org/10.1042/BST20130284

Wardlaw, J. M., Smith, E. E., Biessels, G. J., Cordonnier, C., Fazekas, F., Frayne, R., Lindley, R. I., O'Brien, J. T., Barkhof, F., Benavente, O. R., Black, S. E., Brayne, C., Breteler, M., Chabriat, H., DeCarli, C., de Leeuw, F. E., Doubal, F., Duering, M., Fox, N. C., … Dichgans, M. (2013). Neuroimaging standards for research into small vessel disease and its contribution to ageing and neurodegeneration. *The Lancet Neurology*, *12*(8), 822–838. https://doi.org/10.1016/S1474-4422(13)70124-8

Wen, X., Zhang, H., Li, G., Liu, M., Yin, W., Lin, W., Zhang, J., & Shen, D. (2019). First-year development of modules and hubs in infant brain functional networks. *NeuroImage*, *185*, 222–235. https://doi.org/10.1016/j.neuroimage.2018.10.019

Winters, C., Van Wegen, E. E. H., Daffertshofer, A., & Kwakkel, G. (2015). Generalizability of the Proportional Recovery Model for the Upper Extremity After an Ischemic Stroke. *Neurorehabilitation and Neural Repair*, *29*(7), 614–622. https://doi.org/10.1177/1545968314562115

Xiao, L., Ohayon, D., Mckenzie, I. A., Sinclair-Wilson, A., Wright, J. L., Fudge, A. D., Emery, B., Li, H., & Richardson, W. D. (2016). Rapid production of new oligodendrocytes is required in the earliest stages of motor-skill learning. *Nature Neuroscience*, *19*(9), 1210. https://doi.org/10.1038/nn.4351

Yeganeh Doost, M., De Xivry, J. J. O., Bihin, B., & Vandermeeren, Y. (2017). Two processes in early bimanual motor skill learning. *Frontiers in Human Neuroscience*, *11*, 618. https://doi.org/10.3389/fnhum.2017.00618

Zaaimi, B., Edgley, S. A., Soteropoulos, D. S., & Baker, S. N. (2012). Changes in descending motor pathway connectivity after corticospinal tract lesion in macaque monkey. *Brain : A Journal of Neurology*, *135*(Pt 7), 2277–2289. https://doi.org/10.1093/BRAIN/AWS115

Zhang, H., Schneider, T., Wheeler-Kingshott, C. A., & Alexander, D. C. (2012). NODDI: Practical in vivo neurite orientation dispersion and density imaging of the human brain. *NeuroImage*, *61*(4), 1000–1016. https://doi.org/10.1016/j.neuroimage.2012.03.072
