# Supplementary material for: Hand-Arm Bimanual Intensive Therapy Including Lower Extremities in Infants With Unilateral Cerebral Palsy: A Randomized Clinical Trial
Source: JAMA Netw Open. 2024 Nov 18;7(11):e2445133. doi: 10.1001/jamanetworkopen.2024.45133 (PMC11574690; doi:10.1001/jamanetworkopen.2024.45133)
Supplement: Supplement 2. — eTable 1. Baseline Characteristics of Lost or Excluded Participants eTable 2. Characteristics of the Participants Included in the Analysis, at T0 (Baseline) (n = 46) and T2 (n = 43) eTable 3. YC-PEM Results eAppendix 1. Functional Goals Set for the Entire Sample (n = 48) Grouped by Category, With the Frequency of Each Goal eAppendix 2. Method of Activity Count for Both Upper Limbs eAppendix 3. Description of Content and Organization of Baby HABIT-ILE eAppendix 4. Treatment Characteristics eFigure. Treatment Characteristics: Distribution of Motricity Types for the Upper Extremities and the Lower Extremities, Including the Trunk [file jamanetwopen-e2445133-s002.pdf]

Supplemental Online Content

Carton de Tournai A, Herman E, Ebner-Karestinos D, et al. Hand-Arm Bimanual Intensive Therapy Including Lower Extremities in infants with unilateral cerebral palsy: a randomized clinical trial. *JAMA Netw Open*. 2024;7(11):e2445133. doi:10.1001/jamanetworkopen.2024.45133

eTable 1. Baseline Characteristics of Lost or Excluded Participants

eTable 2. Characteristics of the Participants Included in the Analysis, at T0 (Baseline) (n = 46) and T2 (n = 43)

eTable 3. YC-PEM Results

eAppendix 1. Functional Goals Set for the Entire Sample (n = 48) Grouped by Category, With the Frequency of Each Goal

eAppendix 2. Method of Activity Count for Both Upper Limbs

eAppendix 3. Description of Content and Organization of Baby HABIT-ILE

eAppendix 4. Treatment Characteristics

eFigure. Treatment Characteristics: Distribution of Motricity Types for the Upper Extremities and the Lower Extremities, Including the Trunk

This supplemental material has been provided by the authors to give readers additional information about their work.

eTable 1. Baseline Characteristics of Lost or Excluded Participants

Characteristics	Treatment group (n=2)		Control group (n=3)		
	**Lost at T1 (n=1)**	**Lost at T2 (n=1)**	**Lost at T1 (n=1)**	**Not included in all analyses (n=2)***	
Age, CA (months)	19	15	19	7	8
Gender	Male	Male	Female	Male	Male
Lesion side	Right	Left	Right	Right	Left
Lesion type	Grey matter lesion	Grey matter lesion	Grey matter lesion	Brain maldevelopment	Periventricular lesion
Prematurity (<36 weeks)	No	Yes	No	No	Yes
GMFCS-ER	I	II	I	IV	V
Mini- MACS	I	III	I	V	V

Abbreviations: CA, corrected age; GMFCS-ER, Gross Motor Function Classification System-Expanded and Revised (under 2 years old); Mini-MACS, Manual Ability Classification System for children 1-4 years (scored by clinicians). * Their data could not be analyzed since blind examiners declared them invalid due to no participation.

© 2024 Carton de Tournai A et al. *JAMA Network Open.*

eTable 2. Characteristics of the Participants Included in the Analysis, at T0 (Baseline) (n = 46) and T2 (n = 43)

Characteristics	Categories	Timing	Treatment group (n=24)	Control group (n=22)	Total (n=46)
Age, CA **(Mean ± SD)**	Months	T0	13.29 ± 4.29	13.41 ± 4.00	13.35 ± 4.10
		T2	16.17 ± 4.24	16.41 ± 4.02	16.28 ± 4.09
Gender, **No. (%)**	Male / Female		13 (54) /11 (46)	14 (64) /8 (36)	27 (59) /19 (41)
Lesion side, **No. (%)**	Right / Left		11 (46)/13 (54)	10 (45)/12 (55)	21 (46)/25(54)
Lesion type, **No. (%)**	Brain maldevelopments		2 (8)	2 (9)	4 (9)
	Periventricular lesions		5 (21)	2 (9)	7 (15)
	Grey matter lesions		17 (71)	18 (82)	35 (76)
Prematurity, **No. (%)**	<36 weeks/ ⩾36 weeks		7 (29)/17 (71)	6 (27)/16 (73)	13 (28)/33 (72)
GMFCS-ER, **No. (%)**	I	T0	5 (21)	4 (18)	9 (20)
		T2	7 (32)	8 (38)	15 (35)
	II	T0	7 (29)	7 (32)	14 (30)
		T2	7 (32)	6 (29)	13 (30)
	III	T0	6 (25)	6 (27)	12 (26)
		T2	7 (32)	4 (19)	11 (26)
	IV	T0	5 (21)	3 (14)	8 (17)
		T2	1 (4)	2 (9)	3 (7)
	V	T0	1 (4)	2 (9)	3 (7)
		T2	0	1 (5)	1 (2)
Mini- MACS, **No. (%)**	I	T0	5 (21)	7 (32)	12 (26)
		T2	6 (27)	6 (29)	12 (28)
	II	T0	9 (37)	4 (18)	13 (28)
		T2	9 (41)	5 (24)	14 (33)
	III	T0	8 (33)	8 (36)	16 (35)
		T2	6 (27)	7 (33)	13 (30)
	IV	T0	2 (8)	3 (14)	5 (11)
		T2	1 (4)	3 (14)	4 (9)
	V	T0	0	0	0
		T2	0	0	0

Abbreviations: CA, corrected age; GMFCS-ER, Gross Motor Function Classification System-Expanded and Revised (under 2 years old); Mini-MACS, Manual Ability Classification System for children aged 1-4 years (scored by clinicians). At T2, total n=43 because of the drop-outs.

© 2024 Carton de Tournai A et al. *JAMA Network Open.*

eTable 3. YC-PEM Results (3 pages)

	T0, mean (SD)	T1, mean (SD)	T2, mean (SD)	Two-way RM analysis of variance, two groups x three time			
				Time effect		Time x group interaction	
				df ; F	P (η_p^2)	df ; F	P (η_p^2)
**Home average frequency**							
Control group[b]	6.2 [5.6 ;6.6]	6.1 [5.4 ;6.4]	6.2 [5.9 ;6.4]	df=2	P=0.47		
Treatment group[b]	6.1 [5.7;6.4]	6.1 [5.6;6.3]	6.1 [5.8;6.3]	χ²=1.49			
**Home: percentage of activities**							
Control group	50.5 (1.9)	59.1 (14.6)	62.8 (18.2)	df =2	P<0.001[a]	df =2	P=0.89
Treatment group	46.7 (19.3)	53.5 (19.4)	57.4 (17.2)	F=16.94	(0.28)	F=0.11	(0.00)
**Home: level of involvement**							
Control group	4.0 (0.9)	3.7 (0.8)	3.9 (0.8)	df =2	P=0.01[a]	df =2	P=0.06
Treatment group	3.7 (0.8)	3.7 (0.7)	4.1 (0.7)	F=4.82	(0.10)	F=2.96	(0.07)
**Home: percentage of desire to change**							
Control group[b]	40.0 [24.0;63.0]	38.0 [23.0;62.5]	42.0 [23.0;67.0]	df=2	P=0.19		
Treatment group[b]	39.0 [15.5;66.7]	46.0 [30.2 ;65.7]	24.0 [8.2 ;32.5]	χ²=3.30			
**Home: environmental support**							
Control group	45.3 (24.9)	52.4 (16.1)	50.1 (19.5)	df =1.69	P=0.21	df =1.69	P=0.26
Treatment group	48.0 (31.6)	47.0 (21.0)	55.2 (23.8)	F=1.60	(0.04)	F=1.34	(0.03)
**Home: environmental barriers**							
Control group[b]	0.0 [0.0;8.0]	0.0 [0.0;8.0]	0.0 [0.0;8.0]	df =2	P=0.78		
Treatment group[b]	0.0 [0.0;8.0]	0.0 [0.0;8.0]	0.0 [0.0;8.0]	χ²=0.51			
**Home: environmental helpfulness**							
Control group[b]	98.0 [87.2;100]	92.0 [88.0;100]	92.0 [88.0;99.0]	df =2	P=0.06		
Treatment group[b]	100 [88.2;100]	100 [89.5;100]	96.0 [88.0;100]	χ²=5.59			
**Home: environmental resources**							
Control group[b]	93.0 [80.75;100]	90.0 [84.0;100]	93.0 [88.5;100]	df =2	P=0.65		
Treatment group	100 [80.0;100]	100 [80.0;100]	100 [80.0;100]	χ²=0.87			
**Home: overall environmental support**							
Control group[b]	92.0 [89.1;100]	91.0 [87.5;96.5]	92.0 [84.0;95.7]	df =2	P=0.28		
Treatment group[b]	97.0 [87.0;100]	95.0 [86.0;100]	97.0 [88.5;99.0]	χ²=2.52			
**Daycare/ preschool: average frequency**							
Control group[b]	2.0 [0.0;5.7]	5.0 [0.0;6.0]	5.0 [0.0;6.0]	df =2	P=0.002[a]		
Treatment group[b]	0.0 [0.0;4.3]	4.0 [1.8 ;5.6]	4.7 [0.5 ;6.0]	χ²=12.14			
**Daycare/ preschool: percentage of activities**							
Control group[b]	33.3 [0.0;83.5]	67.0 [0.0;67.0]	67.0 [0.0;67.0]	df =2	P=0.025		
Treatment group[b]	0.0 [0.0 ;67.0]	67.0 [25.5;67.0]	67.0 [6.7 ;100]	χ²=7.40			

© 2024 Carton de Tournai A et al. *JAMA Network Open.*

eTable 3. YC-PEM Results (3 pages)

	T0, mean (SD)	T1, mean (SD)	T2, mean (SD)	Two-way RM analysis of variance, two groups x three time			
				Time effect		Time x group interaction	
				df ; F	P (η_p^2)	df ; F	P (η_p^2)
**Daycare/ preschool: level of involvement**							
Control group[b]	4.3 [3.0;5.0]	5.0 [4.5;5.0]	5.0 [4.0;5.0]	df =2	P=0.05		
Treatment group[b]	3.0 [1.0; 4.9]	3.7 [2.3; 5.0]	5.0 [2.3 ;5.0]	χ²=6.00			
**Daycare/ preschool: percentage of desire to change**							
Control group[b]	0.0 [0.0;67.0]	0.0 [0.0;58.5]	0.0 [0.0;83.5]	df =2	P=0.40		
Treatment group[b]	33.5 [0.0;100]	33.0 [0.0 ;100]	0.0 [0.0 ;67.0]	χ²=1.82			
**Daycare/ preschool: environmental support**							
Control group[b]	19.0 [0.0;44.0]	31.0 [0.0;63.0]	38.0 [19.0;56.0]	df =2	P=0.001[a]		
Treatment group[b]	3.0 [0.0;31.0]	38.0 [0.0;54.5]	37.5 [0.0;54.5]	χ²=13.67			
**Daycare/ preschool: environmental barriers**							
Control group[b]	0.0 [0.0;6.0]	0.0 [0.0;0.0]	0.0 [0.0;0.0]	df =2	P=0.16		
Treatment group[b]	0.0 [0.0;0.0]	0.0 [0.0;0.0]	0.0 [0.0;5.0]	χ²=3.64			
**Daycare/ preschool: environmental helpfulness**							
Control group[b]	100 [89.0;100]	95.5 [88.0;100]	92.0 [84.2;100]	df =2	P=0.57		
Treatment group[b]	100 [95.0;100]	100 [94.5;100]	100 [90.0;100]	χ²=1.12			
**Daycare/ preschool: environmental resources**							
Control group[b]	100 [97.0;100]	100 [100;100]	100 [96.0;100]	df =2	P=0.95		
Treatment group[b]	100 [96.0;100]	98.0 [92.0;100]	100 [86.2;100]	χ²=0.10			
**Daycare/ preschool: overall environmental support**							
Control group[b]	98.0 [90.5;100]	97.5 [92.0;100]	94.0 [90.0;100]	df =2	P=0.55		
Treatment group[b]	98.0 [95.5 ;100]	98.0 [93.5 ;100]	98.0 [87.5;100]	χ²=1.18			
**Community: average frequency**							
Control group	3.0 (1.7)	3.3 (1.3)	3.7 (0.8)	df =1.70	P=0.049[a]	df=1.70	P=0.92
Treatment group	2.7 (1.4)	3.0 (1.7)	3.3 (0.9)	F=3.32	(0.07)	F=0.06	(0.00)
**Community: percentage of activities**							
Control group	37.7 (22.9)	39.2 (18.6)	42.4 (17.1)	df =1.58	P=0.032[a]	df=1.58	P=0.48
Treatment group	32.3 (20.0)	35.1 (21.5)	43.4 (17.6)	F=3.96	(0.08)	F=0.67	(0.01)
**Community: level of involvement**							
Control group[b]	3.7 [2.8;4.1]	4.2 [3.3;4.9]	4.5 [3.1;5.0]	df =2	P=0.055		
Treatment group[b]	3.0 [1.7;5.0]	3.0 [1.7;4.2]	3.1 [2.6 ;4.6]	χ²=5.81			

© 2024 Carton de Tournai A et al. *JAMA Network Open.*

eTable 3. YC-PEM Results (3 pages)

	T0, mean (SD)	T1, mean (SD)	T2, mean (SD)	Two-way RM analysis of variance, two groups x three time			
				Time effect		Time x group interaction	
				df ; F	P (η_p^2)	df ; F	P (η_p^2)
**Community: percentage of desire to change**							
Control group[b]	0.0 [0.0;38.5]	18.0 [0.0;47.5]	13.0 [0.0;31.5]	df =2	P=0.027		
Treatment group[b]	0.0 [0.0;26.5]	9.0 [0.0;51.2]	13.0[0.0;72.5]	χ^2=7.19			
**Community: environmental support**							
Control group	33.6 (21.1)	39.1 (15.0)	32.5 (17.4)	df =2	P=0.012[a]	df=2	P=0.005[a]
Treatment group	25.8 (15.9)	35.7 (22.7)	45.2 (19.8)	F=4.69	(0.10)	F=5.66	(0.12)
**Community: environmental barriers**							
Control group[b]	0.0 [0.0;6.0]	0.0 [0.0;6.0]	0.0 [0.0;0.0]	df =2	P=0.11		
Treatment group[b]	0.0 [0.0;10.5]	0.0 [0.0;12.0]	0.0 [0.0;4.5]	χ^2=4.38			
**Community: environmental helpfullness**							
Control group[b]	97.0 [92.0;100]	93.0 [80.0;97.0]	93.0 [83.0;100]	df =2	P=0.27		
Treatment group[b]	98.0 [89.2;100]	97.0 [90.0;100]	97.0 [89.2;100]	χ^2=2.62			
**Community: environmental resources**							
Control group[b]	100 [90.0;100]	100 [90.0;100]	100 [90.0;100]	df =2	P=0.034		
Treatment group[b]	100 [78.0;100]	100 [86.0;100]	100 [90.0;100]	χ^2=3.524			
**Community: overall environmental support**							
Control group[b]	95.0 [92.0;100]	92.0 [85.0;98.0]	92.0 [87.0;100]	df =2	P=0.53		
Treatment group[b]	95.5 [86.5;100]	92.0 [85.0;100]	95.5 [90.5;100]	χ^2=1.26			

Abbreviations: YC-PEM, Young Children's Participation and Environment Measure; T0, baseline - first assessment time; T1, 1 month after baseline - second assessment time; T2, 3 months after baseline - third assessment time.
[a]Significant value. [b]Non-parametric statistics (Friedman RM analysis of variance on ranks), with median and interquartile range.

© 2024 Carton de Tournai A et al. *JAMA Network Open.*

eAppendix 1. Functional Goals Set for the Entire Sample (n = 48) Grouped by Category, With the Frequency of Each Goal

Goals have been simplified for pooling purposes.

Category	Goal	Frequency
Eating	Drinking from a bottle (2 hands needed because of the weight)	19
	Eating alone with a spoon	16
	Holding the plate while eating alone	6
	Drinking from a cup	6
	Holding and eating a yogurt	3
	Going with the hand to the mouth to eat a biscuit	1
Dressing	Extending the paretic hand for helping putting a t-shirt/ pull/ jacket on	14
	Taking off trousers	3
	Removing socks	2
	Removing a t-shirt	1
	Lowering the pants/diaper	1
	Helping to remove long-sleeved shirts	1
	Unzipping the jacket/coat	1
Displacements	Rolling over (stomach to back or back to stomach)	14
	Crawling to reach a toy (various distances depending on abilities)	11
	Going from lying down to sitting	9
	Riding a baby balance bike	7
	Walking holding on to furniture (to reach toys)	5
	Sitting on a small chair (to play or eat)	4
	Sitting on the ground (to play)	4
	Independent walking (various distances depending on abilities)	4
	Rising from the ground holding a table	3
	Walking on unstable ground (various distances and grounds depending on abilities)	2
	Unsupported standing to play with both hands	2
	Going from sitting to prone	2
	Walking on 4 points to reach a toy (various distances)	2
	Walking holding a doll stroller/ trolley	2
	Less falling when turning (walking)	1
	Raising the head in prone position	1
	Carrying larger objects while walking	1
	Climbing 2 steps to go to parents' bed	1
	Less falling while walking	1
	Going down stairs (seated or 4-points)	1
	Holding the head in a seated position	1
	Climbing into chair with small step	1
	Overtaking a step while crawling	1
	From sitting (chair) to standing	1
	Climbing stairs (4 points)	1
	Standing holding to the chest toy box and pick up toys	1
	Standing alone with front support	1
Playing	Playing "music" with both hands (tambourine, cymbals, maracas)	11
	Opening and turning the pages of a book	8
	Playing with a ball with 2 hands	6

© 2024 Carton de Tournai A et al. *JAMA Network Open.*

	Playing standing in front of a table	4
	Disassemble toys (velcro, duplo)	4
	Playing with 2 hands at nesting cubes	3
	Playing sitting on the floor (small cars, maracas)	2
	Catching objects while lying on the back	2
	Imitating with 2 hands the lullabies	2
	Stabilizing the sheet of paper when drawing	2
	Building lego with 2 hands	2
	Releasing an object with the paretic hand	1
	Starting using painting (handpainting)	1
	Transfering a toy from one hand to the other	1
	Unscrewing a small pot (to put objets in and out)	1
	Holding a large object with 2 hands	1
	Playing with pop beads	1
	Sliding down the slide	1
	Climbing a slide's ladder	1
	Pulling a string to play with a rolling toy	1
	Holding a box with one hand while opening it with the other hand	1
Others	Putting the pacifier in the mouth	2
	Washing both hands	1
	Pressing the toothbrush button	1

© 2024 Carton de Tournai A et al. *JAMA Network Open.*

eAppendix 2. Method of Activity Count for Both Upper Limbs

Participants

To compare activity levels of both upper extremities between the treatment and the control situation, the same 12 infants were included for each condition.

Material

The Xsens DOT® Wearable Sensor, a wearable inertial motion tracker (3D accelerometer, 3D rate gyroscope and 3D magnetometer), was used to evaluate motor activity counts.

Procedure

The infants wore a Xsens DOT® sensor on each wrist during 5 hours a day (maximum recording time at 60Hz of those sensors) during 3 days the week before the HABIT-ILE camp (i.e. during a control situation) and subsequently during the HABIT-ILE camp (treatment intervention). To document inertial measurement units (IMU) data, a daily logbook of activities was completed, by parents for the control intervention condition and by therapist for the treatment intervention condition.

Data analysis

Activity counts (AC) are measures that quantify acceleration within a time unit. 3D IMU 60Hz data were filtered first to remove artifactual transient peaks of acceleration (> +/- 16 g) and secondly with a fourth-order Butterworth bandpass filter to remove the gravity constant component. AC were computed for each dataset as follows: for each dataset the resultant acceleration was segmented in 1-s chunks to obtain the average acceleration per second. Then, AC were obtained by dividing acceleration by the activity count ratio (r =0.001664 g/AC). Finally, AC/s are averaged along each dataset, for each hand and condition (control and treatment) separately.

We included the data of the infant in the analysis if they had a minimum of 1 day of recording in each condition for the 2 sensors. 4 children were excluded from the analyses because parents had difficulties exporting the data each day. Nap times were manually removed in both conditions.

Statistical analysis

After testing the normality of the data distribution with the Shapiro-Wilk test, the AC of both conditions were compared using non-parametric tests (Wilcoxon signed-rank test), for the less affected upper extremity (UE) and the more affected UE separately.

© 2024 Carton de Tournai A et al. *JAMA Network Open.*

eAppendix 3. Description of Content and Organization of Baby HABIT-ILE

Baby HABIT-ILE consists of an adaptation of HABIT-ILE. This intervention was carried out in a camp setting with twelve infants per camp, accompanied by one or two interventionists per child (occupational therapists or physiotherapists), under the supervision of the HABIT-ILE team of experts. The camps were held in a building rented in the middle of a private park usually used for daycare of typically developing children during holidays. The building consisted of a ground floor surface of 120 square meters, divided in two communicating rooms, one including a kitchen space, plus two bathrooms. Full access to the garden outside was provided. Due to COVID-19, a waiting room was set up under a tent, and masks were required at all times for adults. At the initial assessment, conducted within two weeks prior to the start of the camp, the therapeutic team assessed the child and subsequently conducted a semi-structured interview (COPM) to set between four and five functional goals. These goals may be related to activities of daily living such as eating, dressing, moving, playing, or any other functional tasks. The goals must adhere to the SMART criteria, i.e. specific, measurable, achievable, relevant and time bound. The following are illustrative examples: drinking from a bottle (with both hands, as the bottle is too large and heavy to be held with one hand), lowering the pants, turning over in his/her bed (tummy to back or back to tummy), disassemble Duplo's, or wash both hands. In the week preceding the camp, parents were requested to provide videos of their child attempting to achieve the goals in their usual environment. These videos were subsequently analyzed by the supervisory team, and a task analysis was performed. The movements to be sought for the first days of the camp (inclusion of the paretic hand, extension of the elbow, whole hand grasping, trunk stability in standing position in front of a support,…) and the activities to induce these movements were then defined. On the Sunday before the start of the camp, a briefing was held with the interventionists. The camp was conducted over a period of two weeks, from Monday to Friday. The therapy day started at 9am. On the first day of the program, parents were required to stay with their child for at least one to two hours, depending on the child's level of anxiety. It facilitated creating a link between the interventionist, the parent and the child. On other days, the day's activities began with recitation of a nursery rhyme in the presence of the parents (creating some routines in the day decreases anxiety in young children). Subsequently, if the child was at ease, the parents took their leave, thus allowing the interventionist to continue the activities. In the therapy room, each child had their own designated space. The space included various furnishings, including mats, tables, stools, or foam play cushions, selected to align with the abilities and goals of the infant. Interventionists had a whole range of games at their disposal: rattles, balls, games that make music or light, building games, small kitchen games, books, car garage, etc... as well as larger attractive furniture such as a little house, a little shop, a little kitchen, a small slide, a lot of big teddy bears etc... The activities proposed or the way parts are presented to the child must induce the desired movement, with a hands-off concept, which means in infants without guidance of the movement (obviously if the child cannot move by him-herself, the therapist will bring him/her to the bathroom or the kitchen for instance). Around 10:30am, a break of about 30 minutes is allotted for children requiring a nap – naps were systematically managed by the parents. At 11:30, meals are consumed on-site as part of the therapeutic process. Children who who had not napped by 10:30 a.m. completed their morning therapeutic session by 12am, while the others finished by 12:30am. Subsequently, all infants had a period of rest/nap with their parents until 2:30 p.m. Rest/nap times took place either at home or in case parents were far from their accommodation in a building within the same park where each infant spent the nap time with his/her parent. During this time, the interventionists and the supervision team had a team meeting discussing the morning's progress, and the interventionists were given new indications to work on, depending on the progress of the child, to induce a shaping. The afternoon session began and finished (at 4:30pm) with a small song. Finally, at the end of the day, parents had an opportunity to discuss their child's progress with the interventionists and the supervision team.

During the last 3 days of the camp, videos were made of the infants achieving the goals and a report was written to the parents. This report indicated whether the goals had been achieved or were in progress, how the child was achieving the goal (description of the position of the child, the different steps and the adaptations or feedback if needed), and how the child's parents or therapists could continue to train the goals at home or during the conventional therapy. The camp ended with a small ceremony to congratulate all participants, followed by a discussion with parents to talk about progress toward goals.

The unique idea behind baby HABIT-ILE is the sustained stimulation of hand function and gross motor skills. In infants it is very well illustrated in cases where there is no voluntary movement nor attention in one upper extremity. In contrast to an intervention that is solely focused on the upper extremities, infants participating in HABIT-ILE are engaged in a variety of gross-motor activities that facilitate movement in the affected extremity. This approach differs from practice solely focused on upper extremities where infants are installed in a baby relax or highchair, to provide optimal conditions for manipulation. To illustrate, if a child has a goal of sitting independently in his/her bed and for instance a goal of helping to dress by pushig his/her arm in a sleeve, the

environment will be arranged to facilitate repeated play scenarios in which the child is in a semi-recumbent position, enabling him/her to sit with effort through pushing on the affected upper extremity while attempting to grasp a toy with the less-affected upper extremity. While the child is seated freely on a mat, scenarios will be presented in which the child is positioned with large books or toys placed on his/her knees, requiring to remove the affected arm from the toy to use it. While crawling to move around the room towards attractive objects, soft obstacles will be positioned to require the child to crawl over them and to pass his/her arm over it. Such strategies enable the acquisition of movements in the affected upper extremities, which had previously not been observed. This was further refined in accordance with changes observed and the attainment of goals. However, in general, the implication of the upper extremity during gross motor is constant, even in “older" infants. This can be illustrated by the learning to make steps around a table to play, in which the more affected hand/arm was required to manage balance while the other hand was used to play. As some early developmental milestones entail an upper limb component (e.g., sitting arm propping, crawling, four-point stances, pulling oneself up to a standing position and walking while holding on to furniture) the stimulation of gross motor function had a direct impact on the improvements observed on the more-affected upper extremity.

We cannot describe the therapeutic process for each child since each treatment content depends on mainly 3 factors: 1) initial abilities of the child, 2) functional goals set, 3) preferences of the infant him/herself.

However, two illustrative examples of the shaping and evolution of functional goals for two children are presented here.

First, the evolution of a 13-month-old infant, with as goal of reaching out with the more affected hand to assist in putting on a t-shirt. At the beginning of the camp, the child completely neglected her more affected upper limb. Her fist remained closed and her elbow flexed. The first activities proposed were activities aiming to include her more affected upper extremity: removing scrunchies from her more affected upper extremity with her less affected hand, while sitting on a backless bench adapted to her size or sitting on the ground on a mat, then stabilize objects on a table with the more affected hand while removing velcro objects with the less affected hand, sitting on a bench. Once she included more of her more affected hand, activities where she had to extend her elbow in front of her to drop towers, while sitting on a bench, were proposed. Then, activities in which she had to pass her hands through tunnels, first wide and rigid, then narrower and more flexible, to come press a buzzer at the other end, were carried out, seated on a bench. Once sufficient elbow extension had been achieved, t-shirt activities were carried out. At the end of the camp, when the t-shirt sleeve was presented in front of her, she was able to stretch out her more affected elbow to help with dressing.

The following case report presents the progress of an 18-month-old infant whose goal (one out of 5) was to walk 4 steps independently without the use of assistive devices. Before the baby HABIT-ILE camp, this child was able to stand up from the floor with a support (chair or low table). He was able to remove one hand to play but not both while standing in front of a low table. During the first few days of the intervention, he performed a series of standing activities in front of a low table, where he had to take both 2 hands off the table for a brief period (specific toys requiring both hands, like disassembling), without using his pelvis to support himself. Once he had gained static balance, he had to take 1 or 2 steps between two supports, with toys to transport and games that attracted him on both sides, while controlling his balance. The distance between the two supports was gradually increased, and by the end of the camp the child was able to take 5 steps independently.

The decision to have parents not present during the main therapeutic time – while obviously present in many other times of the day as illustrated by the schedule- was motivated by the very successful observations of the same process in toddlers (including those aged 18 months) and by the fact that in Belgium most of the infants are used to childcare (nursery) and parents are absolutely not used to take part in child rehabilitation. In addition, we wanted to allow as many children as possible to participate and didn’t want to bias the sample by selecting solely infants whose parents can dedicate two full weeks (+ assessments) to the study. We completely adhere with the idea that parents as caregivers is optimal if families have the resources to do so, which may not be the case for all infants / children. In addition to the process detailed earlier, a few mothers who were still breastfeeding returned at different times of the day to feed their baby. In term of anxiety, the level of crying was very low and we followed a very strict internal process to avoid infants’ distress (parents were all aware that in case of cries lasting more than a few minutes, they would be called back). However this procedure was only necessary for 2 out of the 23 infants who completed the camp. Those infants requested a “call back” during the two first days.

© 2024 Carton de Tournai A et al. *JAMA Network Open.*

eAppendix 4. Treatment Characteristics

HABIT-ILE being an intervention that constantly and simultaneously stimulates the upper and the lower extremities, with an inclusion of the trunk, 100% of the reported engagement time was dedicated to the upper extremities' actions (i.e. upper extremities category+ 4 limbs actions) and 100% to lower extremities actions (lower extremity category + 4 limbs actions). For the upper extremities, baby HABIT-ILE consisted, on average, of 63.3% gross dexterity, 1.2% of manipulative games, and 23.6% of functional activities. Moreover, 11.8% of the total engagement time was dedicated to the use of all four limbs (crawling, including dragging and 4-points crawling, scoot in sitting, bottom shuffling and self-supported standing). In addition to their involvement in activities using all four limbs, the lower extremities and trunk were used for 9.5% of the intervention time while lying on a mat, 34.5% while sitting on a mat, 19.4% while sitting on a bench, 12.8% while standing, 4.2% while walking, 3.6% while cycling, and 3.9% in transitions. Those proportions are age dependent, with higher reported engagement time in gross dexterity and lying on mat and sitting activities in younger infant compared to older ones who did more manipulative games and standing movements.

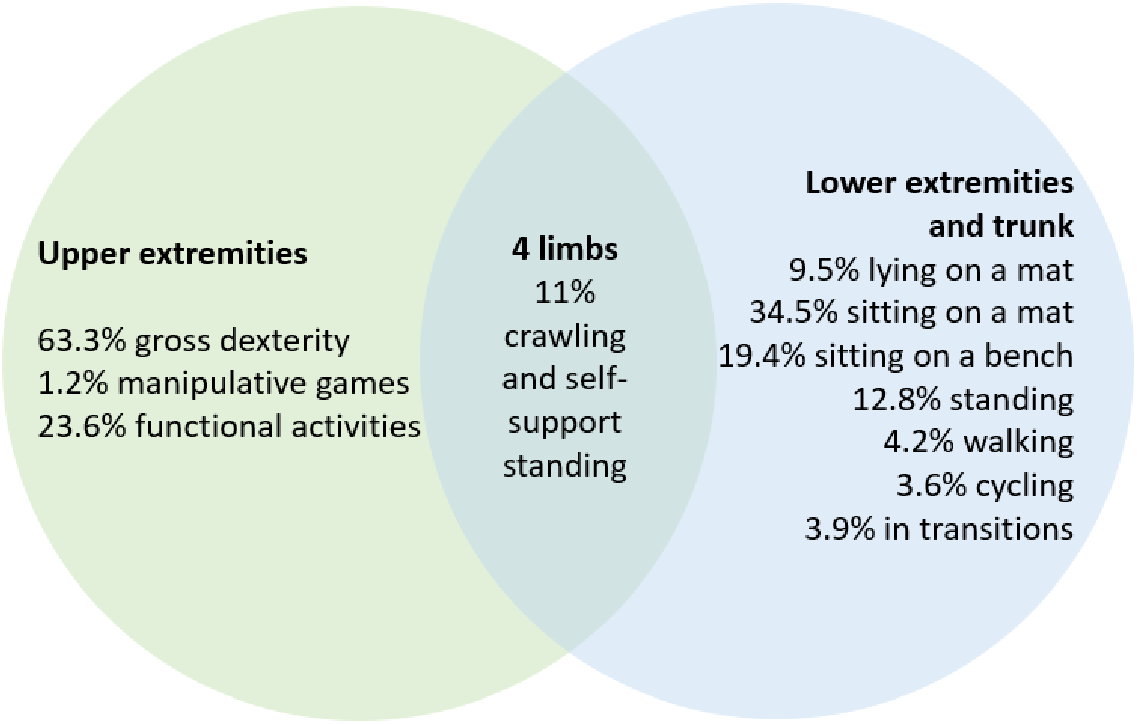

eFigure. Treatment Characteristics: Distribution of Motricity Types for the Upper Extremities and the Lower Extremities, Including the Trunk

© 2024 Carton de Tournai A et al. *JAMA Network Open.*
