# Supplementary material for: Hand-Arm Bimanual Intensive Therapy Including Lower Extremities in Infants With Unilateral Cerebral Palsy: A Randomized Clinical Trial
Source: JAMA Netw Open. 2024 Nov 18;7(11):e2445133. doi: 10.1001/jamanetworkopen.2024.45133 (PMC11574690; doi:10.1001/jamanetworkopen.2024.45133)
Supplement: Supplement 3. — Data Sharing Statement [file jamanetwopen-e2445133-s003.pdf]

Data Sharing Statement

Carton de Tournai. Hand-Arm Bimanual Intensive Therapy Including Lower Extremities in Infants With Unilateral Cerebral Palsy. *JAMA Netw Open*. Published November 18, 2024. doi:10.1001/jamanetworkopen.2024.45133

Data
Additional Information: HABIT-ILE in Infants and Toddlers With Cerebral Palsy (Baby HABIT-ILE) https://clinicaltrials.gov/study/NCT04698395?id=NCT04698395&rank=1 NCT04698395
Data available: Yes
Data types: Participant data with identifiers
How to access data: The data that support the findings of this study will be available upon reasonable request from the corresponding author (yannick.bleyenheuft@uclouvain.be)
When available: With publication

Supporting Documents
Document types: Informed consent form
How to access documents: The data that support the findings of this study will be available upon reasonable request from the corresponding author (yannick.bleyenheuft@uclouvain.be)
When available: With publication

Additional Information
Who can access the data: Researchers whose proposed use of the data will be considered appropiated upon request
Types of analyses: For any purpose
Mechanisms of data availability: After approval of a proposal
